# The Relationship between the Structure of the Tick-Borne Encephalitis Virus Strains and Their Pathogenic Properties

**DOI:** 10.1371/journal.pone.0094946

**Published:** 2014-04-16

**Authors:** Sergei I. Belikov, Ilya G. Kondratov, Ulyana V. Potapova, Galina N. Leonova

**Affiliations:** 1 Limnological Institute, Siberian Branch, Russian Academy of Sciences, Irkutsk, Russia; 2 Research Institute of Epidemiology and Microbiology, Siberian Branch, Russian Academy of Medical Sciences, Vladivostok, Russia; University of Minnesota, United States of America

## Abstract

Tick-borne encephalitis virus (TBEV) is transmitted to vertebrates by taiga or forest ticks through bites, inducing disease of variable severity. The reasons underlying these differences in the severity of the disease are unknown. In order to identify genetic factors affecting the pathogenicity of virus strains, we have sequenced and compared the complete genomes of 34 Far-Eastern subtype (FE) TBEV strains isolated from patients with different disease severity (Primorye, the Russian Far East). We analyzed the complete genomes of 11 human pathogenic strains isolated from the brains of dead patients with the encephalitic form of the disease (Efd), 4 strains from the blood of patients with the febrile form of TBE (Ffd), and 19 strains from patients with the subclinical form of TBE (Sfd). On the phylogenetic tree, pathogenic Efd strains formed two clusters containing the prototype strains, Senzhang and Sofjin, respectively. Sfd strains formed a third separate cluster, including the Oshima strain. The strains that caused the febrile form of the disease did not form a separate cluster. In the viral proteins, we found 198 positions with at least one amino acid residue substitution, of which only 17 amino acid residue substitutions were correlated with the variable pathogenicity of these strains in humans and they authentically differed between the groups. We considered the role of each amino acid substitution and assumed that the deletion of 111 amino acids in the capsid protein in combination with the amino acid substitutions R16K and S45F in the NS3 protease may affect the budding process of viral particles. These changes may be the major reason for the diminished pathogenicity of TBEV strains. We recommend Sfd strains for testing as attenuation vaccine candidates.

## Introduction

Tick-borne encephalitis virus (TBEV) is one of the most dangerous neuroviral infections in humans; transmission occurs via tick bites. Its circulation areas are in the forested zones of many European countries and northern Japan, China, and Mongolia. In Russia, the natural foci of the infection are spread across Kaliningrad Oblast in the west to Sakhalin Island in the east [1]. During the period 1973–2003, there was a 400% increase in the incidence of tick-borne encephalitis (TBE) in Europe [2], [3]. In the 1990s, when the morbidity rate was high, TBE was the cause of at least 11,000 cases of disease in Russia and approximately 3,000 in the rest of Europe [4], [5], [6], [7]. In the 2000s, the incidence of TBE gradually reduced; according to official statistics, Russia recorded only 1,088 cases in 2009 (Federal Service for Supervision of Consumer Rights Protection and Human Welfare (http://rospotrebnadzor.ru/documents/10156/a0a5f062-7498-4af7-b3f6-0a0d7d302c05).

TBEV is a member of the virus genus Flavivirus, of the family Flaviviridae. This genus includes approximately 80 species, many of which are pathogenic in humans, such as West Nile virus, dengue, yellow fever, Japanese encephalitis, etc. [8]. The International Classification of flaviviruses divides TBEV into Far-Eastern (FE), Siberian (SIB), and (Western) European (WE) subtypes [9]. They were named according to their predominant distribution in Eurasia [4], [5], [10], [11], [12].

The clinical course of TBE in the Far East is recognized to be more severe than in other Eurasian areas [13]. The dominant encephalitic form of the disease is characterized by extreme severity and lethality. The main characteristic of FE TBE is the rapid manifestation of overall lesions in the central nervous system, causing focal or diffuse meningoencephalitis involving the brain stem and spinal cord. Until the 1990s, mortality sometimes reached 30% [14]. Since then, the transformation of clinical manifestations of infection in the Far East has been reported to reduce the mortality to 13% and increase the ratio of non-focal TBE forms [13]. This is probably due to the occurrence and spread in the area of new TBEV variants with mutations inducing the subclinical forms of the disease [15].

The exact genetic basis of these differences in the form and severity of the disease is unclear. Previously, the variable severity was associated with the virus subtype [16]. Later, it became clear that there is no direct relationship between the TBEV subtype and severity of the disease. The discrepancy between the virus subtype and the severity of clinical manifestations of the disease is particularly observed in the Russian Far East, where the FE subtype prevails in the absence of SIB and WE TBEV. However, different forms of the disease are registered there, from encephalic forms, which often result in a fatal outcome, to asymptotic and subclinical forms [13]. Previously, many researchers have shown that the pathogenic markers of flaviviruses are localized in the gene of the envelope protein E [17], [18], [19], [20]. Additionally, mutations located in other parts of the viral genome can change the pathogenicity of the virus [21].

As a rule, in order to determine the virulence and pathogenicity of viral strains, their neuroinvasiveness and neurovirulence in mice or changes in the growth characteristics of cell culture have been studied. However, there is no clear correlation between the virulence of strains in mice and strain pathogenicity in humans [22], [23], [24], [25]. Therefore, this requires the definition and analysis of the complete nucleotide sequences of strains isolated from patients with variable severity of the disease for the detection of mutations in the TBEV genome that affect the pathogenicity of strains in humans. The comparison of complete genomes can make it possible to determine previously unknown genome regions that might be correlated with pathogenesis.

In our previous studies, we compared the genomes of several strains isolated from patients with subclinical forms of the disease and indicated specific mutations in strains with different pathogenicity [15]. Nevertheless, it is necessary to analyze more strains to increase the reliability of these results and to exclude single spontaneous mutations that emerged during the evolution of the virus. In this work, we analyzed 34 complete genomes of FE-TBEV strains, which induced variable severity disease in humans. The analysis included the complete genomes of 11 human pathogenic strains isolated from patients with the encephalitic form of the disease (Efd), 19 strains from patients with the subclinical form of the disease (Sfd), 4 Ffd strains with intermediate characteristics, which caused febrile disease in comparison with previously published genomes of the Efd strains Sofjin, Glubinnoe and Senzhang, and a low-virulence strain Oshima 5-10, which was isolated in Japan. We examined mutations in different groups of human Efd and Sfd strains and attempted describe the influence of individual amino acid substitutions in proteins of the virus on the variability of the pathogenicity of TBEV strains.

## Results

### Comparison of nucleotide sequences

The length of the nucleotide sequences of the genomes ranged from 10,405 to 11,103 nucleotides (the conserved region contained 10,376 nucleotides, and the differences were due to the variable length of the 3′ untranslated region (UTR)). Moreover, 8,131 positions were constant in all of the analyzed TBEV strains. Mutations were most often located in the third codon position and were found at 2,245 positions of the genome (21.6%). The aligned sequences of the genomes, 3′ UTR, and polyproteins are not shown, but can be provided upon request. Figure S1 schematically represents the aligned 3′ UTR sequences. A comparison of the complete genome and amino acid sequences of individual viral proteins from all of the sequenced samples and previously reported strains with certain types of human pathogenicity is given in the form of a matrix (Figure S2). It is clear that the complete genomes of the strains differed slightly, and there was no clear boundary between Efd and Sfd strains. Mutations that determine the pathogenicity of strains were difficult to identify on a background of random nucleotide substitutions.

#### Phylogenetic analysis

**Figure 1:**
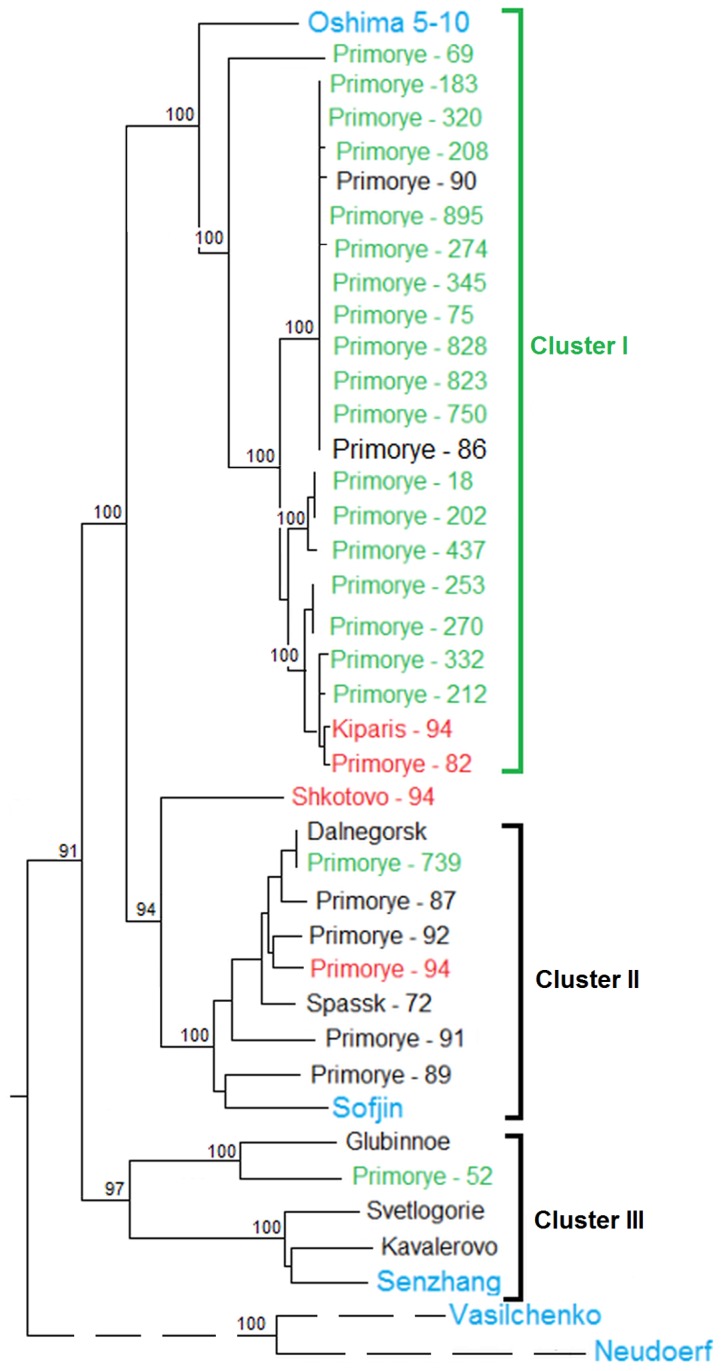
ML phylogenetic tree of TBEV strains. The tree was based on the complete genomes of strains inducing diseases of different severity. Pathogenic strains Efd are shown in black, Sfd strains in green and strains with the febrile form of TBEV are shown in red. Prototype strains are shown in blue. Numbers above or below branches indicate posterior node probabilities and bootstrap values from NJ analysis.

We used maximum likelihood estimation for discrete data analysis to perform phylogenetic analysis of the complete sequenced genomes compared to the previously reported genome sequences of the FE subtype and prototype SIB (Vasilchenko) and WE (Neudoerfl) subtypes of TBEV (Figure 1). The analysis indicates that all of the sequenced strains are related to the FE subtype of the virus and are clearly different from the strains of the SIB and WE subtypes.

The pathogenic Efd strains are divided into two branches, which may be due to the geographical isolation of the speciation areas of the ancestral forms of the virus. Indeed, the strains of cluster II include those related to the Sofjin strain isolated in Russia, Primorye, and the strains of cluster III include those related to the Senzhang strain isolated in northern China. The Sfd strains form a separate cluster (I), but they have a common ancestor with the strains of cluster II. We did not find the Sfd strains to have a common ancestor with those of cluster III. TBEV strains (Fdf) that induced febrile disease did not form a specific separate cluster, except for the Shkotovo-94 strain, which formed a separate branch. Three other strains, i.e. Kiparis-94, Primorye–89, and Primorye–52, induced febrile disease, and are located in clusters II and I. The abnormal location of these strains will be discussed later in the “Exceptions” section.

#### Prediction of secondary structures of genomic 5′ and 3′ non-translated regions and possible motifs of RNA formation

**Figure 2:**
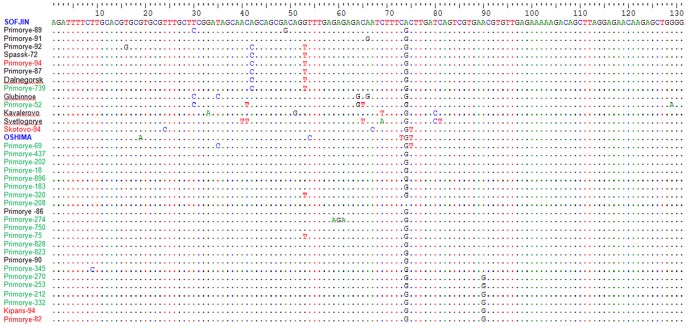
Aligned nucleotide sequences of the 5′ UTR. Pathogenic strains Efd are shown in black, Sfd strains in green and strains with the febrile form of TBEV are shown in red. Prototype strains are shown in blue. The nucleotides identical to the sequence of strain Sofjin are indicated by dots of the corresponding color.

The genomic 5′ non-translated region is conserved and has 131 nucleotides. None of the positions correlated with changes in strain pathogenicity, and single mutations were mainly found in pathogenic strains in the left part of the fragment (Figure 2). In other words, we did not detect a position in which substitutions were reliably different between the Efd and Sfd strains, but were the same within these groups. The predicted 5′ UTR structure of the Sofjin strain consists of three stem loops: a large stem loop (SLA), a second short stem loop (SLB), and a third stem loop (SLC) with an AUG start codon for translation as part of the stem (Figure S3). Previous studies indicated that the SLA region of the dengue virus is a promoter, which is recognized by the viral RNA polymerase RdRp [26]. Despite some insignificant differences in the conformations of the 2D structures, we could not identify any correlation between the specifics of the RNA conformation and strain pathogenicity.

The genomic 3′ UTR is heterogeneous in length, ranging from 31 to 728 nucleotides in different strains, depending on the length of deletions (Figure S1). Although such heterogeneity of the 3′ UTR was reported previously [27], we first described variants of TBEV with extensive deletions covering almost the entire 3′ UTR. Only the pathogenic Svetlogorie strain had a full-length 3′ UTR. The Sfd strains Primorye–69, Primorye–202, Primorye–18 and the Oshima 5-10 strain had a deletion of three nucleotides at positions from 185 to 187 and one nucleotide at position 399. In addition, the pathogenic Primorye–87 strain had two deletions, with three nucleotides removed from positions 185 to 187, and three from 235 to 237. Other analyzed strains had more extensive deletions in the 3′ UTR; their positions and lengths vary in different strains (Figure S1). A characteristic feature of the 3′ UTR of flaviviruses transmitted by ticks is the ∼220 nucleotide deletion from −325 to −545 bp found in many TBEV strains. For example, this deletion is found earlier in strains of the FE subtype isolated in China (MDJ01 (JQ650522), MDJ-02 (JF316708), MDJ-03 (JF316708)), SIB subtype strains (Zausaev (AF527415) and 178-79 (EF469661)), and WE subtype HYPR (U39292). At the same time, we have shown that the position and size of this deletion are not conserved, but vary in different strains (Figure S1). Thus, the deletion is located closer to the stop codon in the Sofjin, Kavalerovo, and Glubinnoe strains (Figure S1).

The first variant strains described with a longest 3′ UTR deletion of 697 nucleotides covering the region from 9 to 705 bp (Figure S1) are the most extraordinary. These strains contain 9 nucleotides near the stop codon and 21 nucleotides in the 3′ UTR terminal portion. Moreover, the exact sequence of the 21-membered terminal fragment is not known, but can be determined by the structure of the primer used for the amplification of RNA. This longest deletion was observed in six Sfd strains: Primorye-208, Primorye-274, Primorye-828, Primorye-823, and Primorye-345, and in two Efd strains Primorye-52 and Primorye-739 located in the cluster of pathogenic strains in the phylogenetic tree, which induced subclinical disease (Figure 1). This suggests that the longest deletion in the 3′ UTR may dramatically reduce pathogenicity.

Most strains, except for those with extensive deletions, and two strains, Spassk-72 and Primorye-437, lacking the terminal portion but with a relatively extended 3′ UTR, have conserved terminal portions of 327 nucleotides in length. This conserved region contains a number of mutations that do not correlate with the pathogenicity of strains and mutations -C245T and -A302G, which differ between groups of strains with different pathogenicity.

The predicted 2D structure of 3′ UTR consists of several loops. The extreme loop within the range from −1 to −85 nucleotides (CR1) is conserved in all strains. Figure S4 shows an example of the predicted 2D structures of the 3′ UTR terminal portion for the Sfd strain Primorye-320 and the pathogenic strain Svetlogorie. Evidently, the topology of the loops does not differ dramatically. Moreover, we show that the -C245T and -A302G mutations, which vary in strain groups with different pathogenicity, are located outside the stems and are probably incidental with no influence on the pathogenicity of strains.

The 5′ UTR and 3′ UTR cyclization disclose the SLB in the 5′ NTR (Figure S5) and part of the loop (CR1) in the 3′ UTR to form a new extended double-stranded fragment (Figure S5), known as the upstream AUG region (5′ UAR) [28]. This fragment is positioned before the AUG start codon and is essential for viral RNA replication [28], [29], [30], [31].

Unlike mosquito-borne flaviviruses, the start codon is located in the SLC, which does not participate in the formation of the 5′ UAR and remains unchanged. The 5′ UTR positions 109–128 were completely conserved in all of the analyzed strains, with no nucleotide substitutions (Figure 2). The 3′ UTR positions −64 to −84 were also conserved in all strains, regardless their pathogenicity, with the exception of strains containing extensive deletions (Figure S1).

Nevertheless, the Spassk-72 strain retained pathogenic properties despite the absence of cyclization motifs.

### Comparison of proteins in TBEV strain groups

Amino acid sequences of polyproteins are more conserved than nucleotide genome sequences. Thus, the observed strain group had only 198 positions (<6.4%) with a substitution of at least one amino acid residue. The polyproteins of pathogenic TBEV (Efd) strains had a length 3414 aa, but Sfd strains had single amino acid deletions in the 111 position of the capsid protein.

Since most amino acid residues in polyproteins are constant, for convenience, we removed the conserved amino acid residues from the analysis and saved only those positions in which we observed at least one amino acid substitution (Figure S6). It is obvious that many single amino acid substitutions are chaotically distributed along the polyprotein. However, there are also substitutions of amino acid residues specific to the Efd and Sfd groups of these virus strains.

Table 1 shows 17 key amino acids, substitutions of which correlate with changes in the pathogenicity of strains. This list includes only those amino acids that are reliably different in strain groups with variable pathogenicity in humans. The table shows that most substitutions of amino acid residues uniquely determine the position of strains in groups with variable pathogenicity, except for a few random substitutions in the strains Sofjin, Primorje-89, Primorye-94, and Kiparis-94. Strains Shkotovo-94, Oshima, and Primorye-69, located at the interface between the Efd and Sfd strains, had an incomplete set of substitutions of key amino acids. In 8 out of 10 TBEV proteins, we found substitutions of key amino acids; they were not found in the non-structural proteins NS2A and NS4A. Substitutions in the two latter proteins did not correlate with changes in the pathogenicity of strains.

**Table 1:** Key amino acid substitutions in pathogenic and unapparent strains of TBEV

	Capsid				prM	E	NS1	NS2B	NS3		NS4B			NS5			
**Strains**	32	69	100	111	151	463	141	108	16	45	95	179	213	634	677	692	724
SOFJIN	**Q**	**K**	**D**	**L**	**A**	**V**	**S**	**F**	**R**	**S**	**M**	**V**	**A**	**S**	**G**	**I**	**A**
**Primorye-89**	•	•	•	•	•	•	•	•	•	•	•	•	•	•	R	•	•
**Primorye-91**	•	•	•	•	•	•	•	•	•	•	•	•	•	•	•	•	•
**Primorye-92**	•	•	•	•	•	•	•	•	•	•	•	•	•	•	•	•	•
**Spassk-72**	•	•	•	•	•	•	•	•	•	•	•	•	•	•	•	•	•
*Primorye-94*	•	•	•	•	•	•	•	•	•	•	•	A	•	•	•	•	•
**Primorye-87**	•	•	•	•	•	•	•	•	•	•	•	•	•	•	•	•	•
**Dalnegorsk**	•	•	•	•	•	•	•	•	•	•	•	•	•	•	•	•	•
Primorye-739	•	•	•	•	•	•	•	•	•	•	•	•	•	•	•	•	•
**Glubinnoe**	•	•	•	M	•	•	•	•	•	•	•	•	•	•	•	•	•
Primorye-52	•	•	•	M	•	•	•	•	•	•	•	•	•	•	•	•	•
**SENZHANG**	•	•	•	V	•	•	•	•	•	•	•	•	•	•	•	•	•
**Kavalerovo**	•	•	•	V	•	•	•	•	•	•	•	•	•	•	•	•	•
**Svetlogorie**	•	•	•	V	•	•	•	•	•	•	•	•	•	•	•	•	•
*Shkotovo-94*	•	•	•	•	•	•	•	•	K	F	•	•	•	•	•	•	•
OSHIMA	R	•	•	V	V	A	•	•	•	F	•	•	•	T	•	V	•
Primorye-69	R	R	•	V	V	A	G	•	K	F	•	•	V	T	K	V	S
Primorye-437	R	R	N	Del	V	A	G	V	K	F	V	A	V	T	K	V	S
Primorye-202	R	R	N	Del	V	A	G	V	K	F	V	A	V	T	K	V	S
Primorye-18	R	R	N	Del	V	A	G	V	K	F	V	A	V	T	K	V	S
Primorye-895	R	R	N	Del	V	A	G	V	K	F	V	A	V	T	K	V	S
Primorye-183	R	R	N	Del	V	A	G	V	K	F	V	A	V	T	K	V	S
Primorye-320	R	R	N	Del	V	A	G	V	K	F	V	A	V	T	K	V	S
Primorye-208	R	R	N	Del	V	A	G	V	K	F	V	A	V	T	K	V	S
**Primorye-86**	R	R	N	Del	V	A	G	V	K	F	V	A	V	T	K	V	S
Primorye-274	R	R	N	Del	V	A	G	V	K	F	V	A	V	T	K	V	S
Primorye-750	R	R	N	Del	V	A	G	V	K	F	V	A	V	T	K	V	S
Primorye-75	R	R	N	Del	V	A	G	V	K	F	V	A	V	T	K	V	S
Primorye-828	R	R	N	Del	V	A	G	V	K	F	V	A	V	T	K	V	S
Primorye-823	R	R	N	Del	V	A	G	V	K	F	V	A	V	T	K	V	S
**Primorye-90**	R	R	N	Del	V	A	G	V	K	F	V	A	V	T	K	V	S
Primorye-345	R	R	N	Del	V	A	G	V	K	F	V	A	V	T	K	V	S
Primorye-270	R	R	N	Del	V	A	G	V	K	F	V	A	V	T	K	V	S
Primorye-253	R	R	N	Del	V	A	G	V	K	F	V	A	V	T	K	V	S
Primorye-212	R	R	N	Del	V	A	G	V	K	F	V	A	V	T	K	V	S
Primorye-332	R	R	N	Del	V	A	G	V	K	F	V	A	V	T	K	V	S
*Kiparis-94*	R	R	N	Del	V	A	G	V	K	F	.	A	V	T	K	V	S
*Primorye-82*	R	R	N	Del	V	A	G	V	K	F	V	A	V	T	K	V	S

Note: the Ffd strains that cause the febrile form of the disease are shown in italics; pathogenic Efd strains are in bold.

• - the same amino acid as in strain Sofjin-HO.

## Discussion

We have sequenced and analyzed the nucleotide sequences of the complete genomes of 34 FE TBEV strains isolated from patients with variable disease severity. The FE TBEV subtype was chosen because its strains normally induce severe disease in humans, which allowed us to collect a sufficient number of strains with diametrically opposite pathogenicity in humans [13].

The severity of TBE may depend on both characteristics of the human host and the state of their immune system, and the specifics of the TBEV genome. In this report, we did not consider the state of the immune system of patients, the location of the tick bite, the exposure time and other factors, which may affect the severity of the disease, but instead focused on the specifics of the genome of TBEV strains isolated from patients with variable disease severity. We previously suggested that specific mutations of the TBEV genome leading to amino acid substitutions could determine differences in disease severity [25]. This study confirms this hypothesis by examining a larger number of TBEV strains. Our study aimed to search for a relationship between the mutations found in the complete genomes of the strains and differences in strain pathogenicity in humans.

There are several reasons for the analysis of complete genomes in a large number of closely related TBEV strains. Spontaneous random single mutations can incorporated into viral RNA during replication by RNA-dependent RNA polymerases and cannot be corrected as the RdRp lack 3′ exonuclease activity. The number of such mutations should increase with an increasing number of passages, if we exclude the possibility of elimination of the viral RNA mutant variants in subsequent passages in another organism of the same species. There is fear that continuous passages of TBEV in suckling mice may have resulted in viral adaptation to mice and induced some of the mutations that were not present in viruses isolated from human cells [32]. This drift had no significant effect in the present study, since we investigated many strains and did not take into account random single mutations; moreover, we only considered the significant differences between the two groups of strains causing disease of varying severity. If specific replacement can be induced during host adaptation, it is logical to assume that it will not be different in the two groups of Efd and Sfd strains, and we did not consider them in our analysis. Another variant is also possible, when a specific substitution occurs in one group of strains. We cannot identify such a change by the present analysis, so it could be mistaken for a key mutation that affects the pathogenicity of strains. Further research could address this question by using reverse genetics, obtaining infectious cDNA clones, introducing point mutations and evaluating neurovirulence in monkeys to detect errors.

Secondly, analysis of strains of only one, and not a few subtypes, eliminates mutations acquired by the strains of different subtypes during a prolonged period of evolution, which can greatly complicate the analysis. For instance, the Sofjin strain of the FE TBEV subtype differs from the Vasilchenko strain of the SIB subtype by 1,486 mutations in the protein-coding domain, resulting in 174 amino acid substitutions. The number of available SIB strains is significantly lower than the number of substitutions; therefore, it is not possible to identify those substitutions that affect the pathogenicity of the virus.

The neuropathogenic potential of TBEV includes two different properties of the virus, i.e. neuroinvasiveness and neurovirulence [33]. We will use the general concept “human pathogenicity” in this report, which is based on the differences in severity of the disease. It should be noted that all analyzed strains, regardless of human pathogenicity, were neuroinvasive and neurovirulent in white mice [25].

Table 2 summarizes the data on the time and place of the isolation of strains, the passage history, and the severity of clinical manifestations. Figure S7 shows a map of Primorye (the Russian Far East) and the locations of strain isolation. It is evident that the majority of Sfd strains were isolated around Vladivostok from persons who visited the forest for short-term domestic purposes or tourism, and then appealed to urban medical facilities for the prevention and treatment of TBE. Residents of remote areas usually do not seek help in such stations, which means that we have no reliable data on circulating strains Sfd strains in these areas.

**Table 2:** Analyzed TBEV strains.

Strains	Passage history^a^	Location, region	Year	Clinical status^b^	GenBank accession #
Sofjin	?	Far east of Russia	1937	Efd	AB062064
Senzhang	?	Northeastern forests of China	1953	Efd	JQ650523
Dalnegorsk	VIII	Dalnegorsky	1973	Efd	FJ402886
Kavalerovo	V	Kavalerovsky	1985	Efd	FJ402885
Spassk-72	VI	Spassky	1972	Efd	JQ825151
Svetlogorie	II	Svetlogorsk	2008	Efd	GU121642
Primorye-86	IX	Kirov	1986	Efd	EU816455
Primorye-87	V	Kavalerovsky	1987	Efd	JQ825149
Primorye-89	VII	Arsenyev	1987	Efd	FJ906622
Primorye-90	VII	Arsenyev	1990	Efd	FJ997899
Primorye-91	VI	Spassky	1991	Efd	JQ825150
Primorye-92	IV	Vladivostok	1992	Efd	HQ201303
Kiparis-94	III	Nadezhdinsky	1994	Efd	JQ825146
Primorye-82	IX	Vladivostok	1982	Ffd	JQ825148
Primorye-94	VI	Nadezhdinsky	1994	Ffd	EU816454
Shkotovo-94	V	Shkotovsky	1994	Ffd	JQ825147
Oshima 5-10	?	Kamiiso, Hokkaido	1995	-	AB062063
Primorye-18	V	Vladivostok	1997	Sfd	GQ228395
Primorye-52	V	Anisimovka	1999	Sfd	JQ825154
Primorye-69	VI	Ussuriysk	2000	Sfd	EU816453
Primorye-75	II	Nadezhdinsky	1999	Sfd	JQ825152
Primorye-183	IV	Nadezhdinsky	1991	Sfd	JQ825153
Primorye-202	VI	Nadezhdinsky	1997	Sfd	JQ825157
Primorye-208	VII	Lazurnaya	1991	Sfd	JQ825158
Primorye-212	II	Vladivostok	1991	Sfd	EU816450
Primorye-253	II	Nadezhdinsky	1991	Sfd	EU816451
Primorye-270	III	Nadezhdinsky	1991	Sfd	EU816452
Primorye-274	III	Nadezhdinsky	1999	Sfd	JQ825159
Primorye-320	II	Shkotovsky	1999	Sfd	JQ825160
Primorye-332	V	Nadezhdinsky	1991	Sfd	AY169390
Primorye-345	V	Vladivostok	1999	Sfd	J Q825161
Primorye-437	VIII	Lazurnaya	1999	Sfd	JQ825162
Primorye-739	IV	Vladivostok	1992	Sfd	JQ825156
Primorye-750	III	Kiparisovo	1998	Sfd	JQ825163
Primorye-823	II	*Krasnoarmeisk*	2000	Sfd	JQ825164
Primorye-828	IV	Chernigovka	1998	Sfd	JQ825144
Primorye-895	II	Vladivostok	2000	Sfd	JQ825145

a suckling mouse brain.

b Efd, encephalitis form of disease; Ffd, febrile form; IF, Sfd, subclinical form.

Note: “?” passage history not available

### Analysis of nucleotide sequences

The length of virus RNA of the analyzed strains ranged from 10,404 kb (Primorje-208) to 11,103 kb (Svetlogorie), depending on the size of the deletion in the 3 ′NTR. Figure S2 shows the data on amino acid and nucleotide sequence similarities of conservative parts of the genomes. Evidently, there is no clear boundary between the virus strains, which induced variable severity of the disease. The values of synonymous (*d*_S_) and non-synonymous (*d*_N_) substitution rates calculated for the complete coding region of the genome by codeml in the PAML package [34], [35] were low. The values *d*_N_/*d*_S_ (max)  = 0.0615 and *d*_N_/*d*_S_ (mean)  = 0.0416 indicate no adaptive selection in the analyzed sample group of strains, which corresponds to previously reported data on other flaviviruses [36], [37].

There are three clearly visible clusters in the phylogenetic tree (Figure 1). Cluster I mainly contained Sfd strains and the Oshima strain, which was isolated in Japan (its pathogenicity in humans has not been described), while clusters II and III contained human pathogenic Efd strains, including the prototype Sofjin [38], [39] and Senzhang [40] strains which were previously isolated in the Russian Far East and northeast China, respectively. The strains in TBEV clusters II and I have a common ancestor, while the clusters I of Sfd strains are younger. There was no separate group of Ffd strains identified, which was able to induce the febrile form of the disease, except for the Shkotovo-94 strain, allocated between II and I clusters (Figure 1). The three other strains which resulted in febrile TBEV were either among the pathogenic strains of cluster II (Primorye-94) or the Sfd strains of cluster I (Kiparis-94 and Primorye-82). The specifics of these and some other strains whose position in the phylogenetic tree did not coincide with the severity of disease (Primorye-86 Primorye-90, Primorye-52 and Primorye-739) are described in the special section “Exceptions”.

The specifics of the 5′ and 3′ UTR sequences may change TBEV pathogenicity [41], [42]. The genome regions capable of forming complementary complexes between the 5′ UTR and 3′ UTR fragments (so-called cyclization domains), which are necessary for the replication of viral RNA, play an important role in TBEV replication [26], [29], [43], [44]. The figures S5 show that the secondary UTR structures of Efd strain Svetlogorie and Sfd strain Primorye-18 both have a full-length 3′ UTR. It is clear that there are no significant differences in the structures. The sequences forming complementary complexes between the 5′ UTR and 3′ UTR fragments are conserved across all strains, regardless of their pathogenicity. In the sequences of the 3′ core element of the analyzed strains, we found several single nucleotide substitutions, three of which (A426G, C483T and G532A/C) were different in Efd and Sfd strains, but these do not affect the two-dimensional packaging of the 3′ UTR (Figure S4).

Thus, mutations detected in the 5′ UTR and 3′ UTR sequences of Efd and Sfd strains do not correlate with pathogenicity and are likely to be random. Furthermore, mutations in the 3′ UTR, including those that differ between Efd and Sfd strains, do not affect the pathogenicity and are random. However, it is possible that unidentified specific nucleotide sequences in the 3′ UTR can affect the position and size of deletions that occur when the virus is transmitted from tick cells to mammalian cells.

One feature of the 3′ UTR in the analyzed strains is the presence of extended deletions that vary with respect to position and size across strains. The mechanism of the occurrence of these deletions, their role and their importance to the evolution of the viral population are uncertain; hence, this problem requires a detailed discussion. Previous studies have shown a different degree of influence from the artificial introduction of extended deletions of different lengths, starting from the stop codon to the core element, on the survival and pathogenicity of viral strains [45], [46].

Analysis of our data also indicates that extended deletions have no significant effect on the pathogenicity of the strain and the efficiency of virus replication in mammalian cells, if they do not affect the conservative end portion of the 3′ UTR of approximately 325 nucleotides. This region of the 3′ UTR includes regulatory elements and sequences involved in the cyclization of viral RNA (Figure S1). If this suggestion is correct, and the presence of extended deletions in the 3′ UTR does not affect the core element and replication of the virus, the existence of virus variants with a full-length 3′ UTR in nature is unclear, as such variants should be eliminated from the virus population within several cycles of host changes. However, it is possible that this elimination does not occur because a removable fragment of the genome in mammalian cells is significant for the survival of TBEV in tick cells [10], [27], [47].

TBEV is known to circulate in nature by cyclic transmission from ticks to small mammals, followed by return to the ixodid tick vector, where the virus propagates and persists for a long period. The stage of virus survival in mammalian cells is necessary, but insignificant in terms of time [48], [49]. The fragment of the viral genome that is removed in mammalian cells cannot reappear in tick cells during alternating cycles of virus transmission from ticks to mammals, and again to ticks. This fact may indicate that removal of the 3′ UTR fragment is not quantitative; therefore, some part of the viral population in cells of mammals has a full-length 3′ UTR. Perhaps only that viral particle containing the full-length RNA can be effectively transmitted to ticks feeding on an infected animal. Variants of the virus that have lost the 3′ UTR fragment during reproduction in mammalian cells probably cannot reproduce effectively in tick cells, and probably cannot participate in the circulation of the virus in nature.

The studies by Labuda et al. confirm this suggestion. They showed that TBEV is most effectively transmitted from infected to naïve ticks during co-feeding on an animal [50], [51]. Labuda and co-authors showed that viremia or systematic infection are not necessary for successful transfer. Moreover, they may impede the transmission of the virus. Our suggestions on the structure-mediated removal of the 3′ UTR fragment affected by unknown enzymes of mammalian cells, and the inability to transmit viral particles with the 3′ UTR fragment removed in the tick population, correspond well with these data. The mechanism behind fragment removal and the connection between the size and location of deletions with the nucleotide sequences of the fragments are unknown. It is possible that the rate of removal of the 3′ UTR fragment from the viral genome depends on the cell type and is minimal in dendritic cells, which are the primary targets of the virus [52]. Labuda et al. did not study the 3′ UTR structure in their work on the virus transmission between co-feeding ticks; it would therefore be interesting to repeat these studies with the identification of deletions at the 3′ UTR and the possible effect of deletions on the transmission of the virus in the tick population.

A terminal core element with highly conserved nucleotide sequences was previously found in all analyzed TBEV strains. This conserved site has been indicated in many publications [27]. The entire core element was predetermined to form a well-defined secondary structure, depending on the sequences of the neighboring variable elements of the genome [53]. Most of the analyzed strains have a terminal conserved core element, of about 325 nucleotides in length, except for the three Efd and six Sfd strains that lack the core element. Previously, Mandl et al. showed that mutant variants of the virus with the deletion covering the core element are non-viable [45]. In our study, the survival of strains with deleted core elements was apparently determined by incomplete (non-quantitative) removal of the 3′ UTR fragment. Such a mixture of variants with full-length and deleted 3′ UTRs is able to survive in vertebrates, but our method of analysis of the 3′ UTR sequence may not accurately determine the number of different virus variants.

We identified similar strains with the extended deletion in the group of Sfd strains (Primorye-208, Primorye-274, Primorye-345, Primorye-437, Primorye-823 and Primorye-828) and in the group of Efd strains (Primorye-52 and Primorye-739). The latter two strains induced a subclinical form of the disease; hence, an extensive deletion in the 3′ UTR dramatically reduces the pathogenicity of the strains in humans. We suggest that the extensive deletion in the 3′ UTR in the Primorye-739 and Primorye-52 strains probably occurred during the initial stages of infection in patients, which resulted in a reduction in the pathogenicity of the virus and a subclinical course of the disease.

The Spassk-72 strain, which is located in a cluster of pathogenic strains and has an unusual extended deletion in the 3′ UTR, including the core element, was isolated from a patient with fatal encephalitis. This may be due to the content in the human body of a considerable amount of virus variants with a full-length 3′ UTR, which was not observed by us.

Unfortunately, our method of genome sequencing is not suitable for the quantitative determination of the ratio of full-length and deleted variants of the virus; therefore, it is necessary to develop a novel approach to the analysis of 3′ UTR variability during the transmission of TBEV from ticks to different mammalian cells. It is possible that the recently reported approach based on using a circular polymerase extension cloning (CPEC) reaction can be adapted [54].

### Analysis of amino acid sequences

The viral replication cycle includes several stages, i.e. attachment of the virus to the surface of the target cell, RNA replication, and synthesis and processing of the viral protein and assembly of viral particles [55].

**Figure 3:**
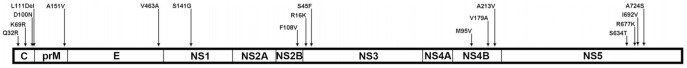
Tertiary structure of NS3 protease. The crystal structure of the West Nile protease (PDB code 3e90) was taken as a template for homology modeling. (A) The tertiary structure of NS3/NS2B for the pathogenic strain Dalnegorsk. (B) The tertiary structure of NS3/NS2B for the Sfd strain Primorye-270. The arrows indicate the replacement of key amino acids. The active center is indicated by the rectangle and its amino acid residues are shown as red atoms.

Amino acid substitutions in various parts of the polyprotein can influence different stages of viral replication and strain pathogenicity, but the exact mechanisms behind this influence are unknown. The role of each amino acid is also unclear. Most amino acid substitutions found in the viral population may be incidental and (or) determined by the adaptation of the virus to various environments, meaning that they cannot affect the pathogenicity of strains. We found 198 positions with substitutions of the amino acids in the polyprotein with a length of 3414 aa (Figure S6). The substitutions at most positions were specific to small strain groups, which characterize the evolution of the viral population, rather than correlating with changes in the virulence of strains. However, 17 amino acid substitutions were different between the Efd and Sfd groups of strains (Table 1). Table 1 shows that both structural and non-structural proteins had specific amino acid substitutions and Figure 3 indicates the position of the specific key amino acid substitutions in the polyprotein.

Below, the function of each protein and the possible role of key amino acid substitutions are provided in more detail.

Viral particles of all flaviviruses have a nucleocapsid surrounded by a lipid bilayer membrane, inside of which there are two fixed structural proteins, the surface protein, E (approximately 54 kDa), and precursor membrane protein, prM (approximately 27 kDa) [8].

Cleavage of the capsid protein from the polyprotein occurs in two stages:

- In the first stage, the cleavage of C-prM, by host signalases, forms a membrane-bound protein C with a signal peptide (C_int_).

- The second stage is the cleavage of the membrane-bound form of protein C_int_ by the viral protease, the NS2A/NS3 complex, forming the mature capsid protein [56]. However, a previous study showed that the cleavage of the C-prM bond occurs inefficiently in the absence of the viral protease, and initially cleaves the signal peptide from C_int_ [57].

Despite these different hypotheses, the efficient assembly of viral particles requires a strict balance of the catalytic activities of the two proteases, i.e. the signalase and the viral protease [58]. The nucleocapsid is capable of integrating into the bud formed by the surface proteins prM and E in the membrane of the endoplasmic reticulum. With the accumulation of 180 prM and E proteins, this bud is separated from the parental membrane, and the immature viral particle is transported from the cell through the trans-Golgi network. In the absence of the nucleocapsid, proteins prM and E with part of the membrane can efficiently form small capsid-free subviral particles (SVPs) [59]. Recent studies on TBEV showed that the introduction of certain deletions and amino acid substitutions into protein C disrupts the assembly of infectious particles, resulting in the formation of many non-infectious subviral particles [53], [60], [61].

Structural protein C of the studied strains is the most inconstant, i.e. it had three key amino acid substitutions and a deletion of one amino acid at position 111 in Sfd strains, with a protein length of 116 aa, in contrast to Efd strains, which carry the amino acid residues Leu, Val, Met or Ile at this position (Table 1). This deletion is located in the signal peptide, close to the site of signalase cleavage. In addition, the signal peptide contains the substitution D100N in the transmembrane domain. Both of these substitutions can determine the position and effectiveness of processing (i.e. they are contained within the signal peptide cleavage site). Calculations of the most probable cleavage sites indicated that the amino acid deletion does not change the position of the C-prM signalase cleavage site, but can affect the rate of the processing, because the calculated probable cleavage in Sfd strains is significantly lower than in pathogenic strains (Figure S8). The most significant decrease in cleavage site score values was observed for the hypothetical variant having a deletion at aa position 111, but not containing the compensatory substitution D100N (Table S1).

Moreover, we observed two specific substitutions (Q32R and K69R) in the capsid protein of Sfd strains that can affect the packing density of the capsid protein (Figure S9), but these are unlikely to have a significant effect on the pathogenicity of strains. Another substitution, K64N, which was found not only in all Sfd strains, but also in six strains of cluster III on the phylogenetic tree, may also be considered to not affect strain pathogenicity.

The other structural proteins prM and E both contained specific substitutions (A151V and V463A, respectively), which are located in the transmembrane domains and differ between Efd and Sfd strains (Table 1). These amino acid substitutions are conserved and do not significantly affect the conformation of proteins (Figure S10) or the pathogenicity of strains. Single incidental substitutions in the prM protein are outside the sites of protein processing by furin; hence, they are not essential for the function of the protein. In protein E, we also found single conserved amino acid substitutions and substitutions in four small groups of Efd and Sfd strains (Figure S6). These substitutions may affect the immunogenic properties of the virus, but they did not correlate with the pathogenicity of strains.

Thus, only one protein from the group of structural proteins had amino acid substitutions that correlated with the Efd and Sfd groups and may influence the pathogenicity of strains. Substitutions in the transmembrane domain of the capsid protein may affect strain pathogenicity at the budding stage through the formation of capsidless subviral particles. The structural proteins prM and E did not have substitutions that could affect the pathogenicity of the analyzed strains; therefore, the attachment of viral particles to the cells or viral penetration into cells will probably not significantly influenced the pathogenicity of viral strains.

The non-structural protein NS1 is a highly conserved glycoprotein with 12 invariant cysteine residues. Unlike other non-structural proteins, NS1 is secreted and accumulates in the serum of infected subjects [62]. In the infected cell, NS1 serves as a cofactor for viral RNA replication ([63] and references therein) and exists as a dimer [64], [65]. Secreted NS1 forms oligomeric states, up to the hexamer level [66], and participates in complement activation [67], the generation of autoimmune antibodies and the destruction of endothelial cells [68]; however, the exact function of secreted NS1 is unclear [69]. In the NS1 protein of the analyzed strains, we found several single amino acid substitutions and substitutions in two small groups of Efd and Sfd strains that did not correlate with changes in pathogenicity. We also found a key substitution, S141G, which evidently differed between groups of strains with variable pathogenicity (Figure S6). There are no data on the influence of this amino acid on the properties of the NS1 protein; therefore, we cannot assess the role of this substitution in the pathogenesis of TBEV.

The non-structural protein NS2A is a small membrane-bound protein that participates in RNA replication by binding to the 3′ UTR with the proteins of the replicative complex [70]. Furthermore, NS2A inhibits the induction of cellular alpha/beta interferon [71], [72], [73] and is involved in the assembly of viral particles [74,74]. The studied strains contained several amino acid substitutions in this protein, which did not correlate, with changes in the pathogenicity of the strains.

The non-structural protein NS2B is a small membrane-bound protein that forms a complex with viral protein NS3, which has protease catalytic activity. Substitutions in the hydrophilic part of the protein affect the catalytic activity of the complex [75]. The analyzed Sfd strains had a substitution (F108V) located in the transmembrane domain (Figure S10), which did not significantly influence the conformation of the protein and the pathogenicity of strains.

**Figure 4:**
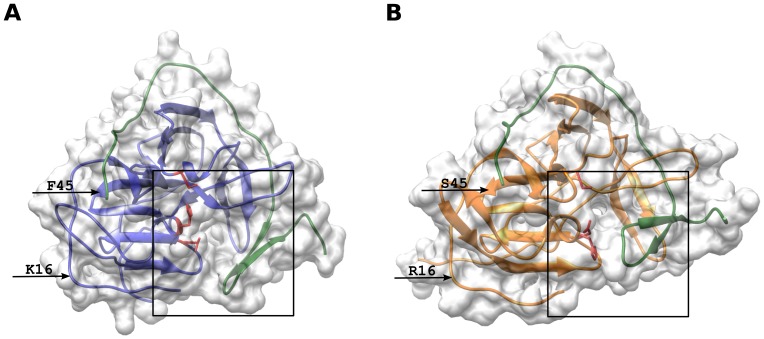
Tertiary structures of RNA-dependent RNA polymerase (RdRp). (A) The tertiary structure of RdRp for the pathogenic strain Dalnegorsk. (B) The tertiary structure of RdRp for the Sfd strain Primorye-270. In both models, the various subdomains are colored as follows: blue – fingers, green – palm, and pink – thumb. The amino acid residue substitutions in both models are indicated by red atoms.

The non-structural protein NS3 is a 70-kDa soluble protein with protease, helicase, NTPase and 5′-terminal RNA triphosphatase activities [76], [77], [78]. N-terminal residues were identified as the serine proteinase domain of the trypsin family containing the residues His-54, Asp-78 and Ser-138 in the active center [79], [80]. Sfd strains possessed two key substitutions, R16K and S45F in the NS3 protein, located in the protease domain, but outside the active center. Molecular dynamic simulations indicated that changes in the conformation of the NS3/NS2B complex in Sfd strains might hinder the formation of the polyprotein/protease complex and reduce the hydrolysis rate of the polyprotein [81] (Figure 4). The biological properties of the Skotovo-94 strain confirm these calculations. The pathogenic properties of this strain are reduced in comparison with pathogenic strains, since the Skotovo-94 strain causes the febrile form of disease. This strain has 15 key amino acids that are specific to pathogenic strains but does not contain the substitutions R16K and S45F in the NS3 protein specific to Sfd strains (Table 1). It is possible that the Skotovo-94 strain induced febrile disease due to the presence of these substitutions in the protease. Skotovo-94 exhibits reduced pathogenicity; however, this reduction is less significant than in typical Sfd strains.

The non-structural protein NS4A is a membrane protein with four transmembrane domains. Moreover, the C-terminal site, denoted as a 2K fragment, serves as a signal sequence for the translocation of the protein NS4B into the lumen of the endoplasmic reticulum ([82], [83] and references therein). The studied strains had several insignificant mutations, which did not correlate with pathogenicity.

NS4B is the largest of the small hydrophobic proteins of the virus and has 252 amino acids and 5 hydrophobic regions with four transmembrane domains (Figure S10). NS4B is known to interact with the helicase domain of NS3 [84] and may serve as an interferon antagonist ([85]; for review, see [86]). The Sfd strains possessed three key substitutions in NS4B, two of which, M95V and V179A, are located in transmembrane domains 2 and 3, while the substitution A213V is located in the hydrophilic area, between transmembrane domains 3 and 4 (Figure S10). The functions of these substitutions are unknown, so it is unclear whether they influence the pathogenicity of these strains.

The protein NS5 is the largest (900 aa) and the most conserved among the flaviviruses. It has RNA-dependent RNA polymerase and methyltransferase activities [86], [87], [88]. Recently, NS5 was reported to inhibit the production of interferon [89], [90], [91], [92].

**Figure 5:**
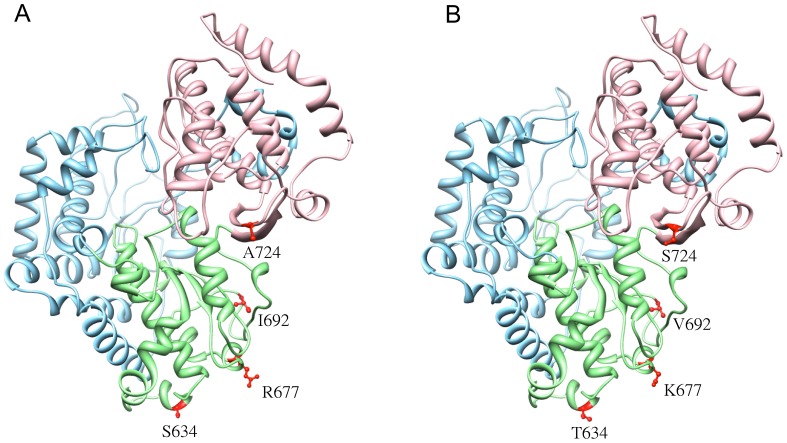
The scheme of the polyprotein and the position of key amino acid substitutions.

The NS5 protein of the Sfd strains contained the key substitutions S634T, R677K, I692V and A724S, which may affect the pathogenicity of TBEV. These substitutions are located in the RNA-dependent RNA polymerase domain (RdRp) of NS5 (Figure 5, Figure S11). The substitution A724S is located in the E motif of the RNA-dependent polymerase, close to an important functional domain that is responsible for binding zinc ions (Zn^2^) [36], while the other three substitutions, S634T, R677K and I693V, are conserved. The substitution I692V is located in the flavivirus conserved domain LNDMAKTRKDI, like in Powassan virus, which is not significantly pathogenic in humans. Other amino acid substitutions were located outside the conserved domains on the palm of NS5, and were geometrically approximated (Figure 5). A combination of several substitutions may have an effect on polymerase activity and may affect the pathogenicity of strains. The key substitutions detected in the present study are likely to affect the inhibition of interferon-stimulated JAK-STAT signaling, since the JAK-STAT inhibitory domain is located between residues 355 and 735 within the RdRP domain [93].

Amino acid substitutions in the NS5 protein can affect the neurovirulence of strains not only through changes in the efficiency of replication of viral RNA or inhibiting of interferon production, but also via interaction with certain cellular proteins which may affect pathogenic properties of the TBEV. Thus, recently published paper indicated [94], based on the substitution of the genome regions of the OHFV strain by the homologous regions of TBEV, that the highest influence (89,9%) on the manifestation of the neurological disease in mice have hybrid strains containing the genome region encoding the protein NS5 of TBEV. A region encoding the protein NS3 affects to a lesser extent (45.5%) [94]. Moreover, it was shown that the C-terminal region of the protein, including the replacement of 4 amino acids 879RYS881 and 891E, characteristic for the OHFV; 879KFK881 and 891D, characteristic for the TBEV has the highest influence on neurovirulence in mice. The authors suggested that KFK-D motif of TBEV affects axonal growth [94]. Notably, the substitution of the site (nt 9488–10295 or aa 608–877) significantly reduced virulence, because most infected mice did not show any signs of disease.

All TBEV strains analyzed contained KFK-D motifs in the protein NS5 (except for several strains having substitutions K/R) and caused neurological disease in white mice, but had different neurovirulence for humans. These facts confirm our previous data on the absence of unequivocal correlation between neurovirulence of strains in mice and humans [25]. Substitutions of the key amino acids, which are different in Efd and Sfd strains (aa 634–724), are located in the middle portion of the NS5 protein. They may reduce the virulence due to the influence on the replication efficiency [94]. These data allow us to conclude the possible impact of key substitutions in the protein NS5 on the severity of the disease, despite the absence of unequivocal correlation between neurovirulence of TBEV strains in mice and humans, and that the spectrum of amino acid substitutions may vary. It is also necessary to indicate the way by which the NS3 protein may influence the development of neurological symptoms.

## Exceptions

We have shown that the strain position on the phylogenetic tree and the presence of certain key amino acid substitutions correlates well with the pathogenicity of TBEV strains. As a rule, the strains of cluster I were isolated from persons with subclinical course of the disease, whereas the strains of clusters II and III caused the severe fatal form of encephalitis. However, there were several exceptions to this rule, some of which can be explained by a more detailed study.

The most rigorous exceptions to this rule are the strains Primorje-86 and Primorje-90, which are Sfd strains based on their molecular characteristics, but were isolated from individuals who had a posthumous diagnosis of “encephalitis” in 1986 and 1990. We have no way to explain this discrepancy, beyond a speculation on the inaccuracy of the diagnosis or the presence of severe immunodeficiency or other comorbidities that could cause an adverse outcome on the background of infection by Sfd strains of TBEV.

The Primorye-52 and Primorye-739 strains caused a subclinical form of the disease, despite the fact that they are located in clusters II and III on the phylogenetic tree and have key amino acids specific to pathogenic Efd strains. The reason for this discrepancy may be the presence of the longest deletion in the 3′ UTR, including the entire core element, which hinders the effective propagation of the virus in human cells. Thus, this exception confirms our suggestion that removal of the longest fragment in the 3′ UTR may significantly reduce the pathogenicity of TBEV strains.

Other exceptions are the strains that cause the febrile form of the disease, which results in the recovery of patients and which can considered as a weakened form of encephalitis. The Shkotovo strain forms a separate branch of the phylogenetic tree between the Efd and Sfd strains and is different from the Efd strains by the presence of the key amino acids 16K and 45F in the viral protease NS3, which are characteristic of the Sfd strains. This indicates that the substitutions 16K and 45F in the viral protease NS3 may reduce the pathogenicity of these strains.

The Ffd strain Primorye-94 had a V179A substitution in NS4B, which is specific to the Sfd strains, but its role in reducing the pathogenicity of strains is unknown. Additionally, this strain has many other unique amino acid substitutions: V141I in protein E, K47R in protein NS1, L69F, M161I and R228K in NS2B, A68V and L143I in NS3, and A92V in NS4A. It is likely that a combination of this set of substitutions reduces the pathogenicity of the strain, but there is no direct evidence of the impact of these changes on pathogenicity.

The strains Primorye-82 and Kiparis-94 represent Sfd strains, but with increased virulence, as they caused the febrile form of the disease. Each strain has several single amino acid substitutions at different sites of the polyprotein (Figure S6). At the same time, both strains showed a unique substitution, D809N, located in the thumb domain of NS5. Additional studies using reverse genetics methods are needed to assess the role of replacement D809N in changing the pathogenicity of strains; this should be evaluated using the monkey safety test for neurovirulence [95].

## Conclusion

Thus, deletions in the structural capsid protein and amino acid substitutions in the non-structural proteins NS3 and NS5 may reduce the virulence of TBEV via effects on viral RNA replication, polyprotein processing and the assembly of viral particles. Amino acid substitutions found in the E protein did not correlate with the degree of pathogenicity of TBEV strains and probably do not affect virus attachment to cells. Substitutions in the 3′ UTR do not play a significant role in changing the pathogenicity of strains, but more research is needed to understand the mechanism of these deletions, their extent and position.

Identifying the role and importance of each key amino acid substitution in the pathogenicity of TBEV strains requires additional studies using site-directed mutagenesis techniques with a full-length infectious cDNA clone. However, this type of work requires a biosafety level 4 laboratory and non-human primates to test the neurovirulence potential of new virus variants.

At the same time, Sfd strains can be recommended for testing as candidates of the live attenuated vaccine without additional modifications. These strains can be divided into 3 groups according to the presence of specific substitutions in the E protein and four groups according to the presence and the length of the deletions in the 3′ UTR.

## Materials and Methods

### Ethics statement

Mouse experiments were carried out in strict accordance with the recommendations in the Guide for the Care and Use of Laboratory Animals according to the USSR Ministry of Public Health Regulation No. 1189 of 10.10.1983. Experiments were performed according to the USSR Ministry of Public Health Regulations No. 755 of 12.09.1977 and No. 701 of 27.07.1978 for the humane treatment of animals. All experimental procedures were approved and performed according to the Research Institute of Epidemiology and Microbiology of the Siberian Branch of RAMS (Vladivostok) Animal Care and Use Committee (protocol # 1 on 30.01.2008).

### Virus isolation

The isolation of TBEV strains was performed as previously described [25]. Briefly, for the isolation Efd strains, autopsy materials (brains) from dead patients were used, while for the Sfd and Ffd strains, fresh blood from a vein was used for virus isolation. All of the strains were isolated by intracerebral injection in 2-day old inbred mice, each with 10 µL of a 10% bioassay suspension. Monitoring the clinical manifestations of infection was performed by daily observations of disease in animals over a period of 2–3 weeks. The brains of deceased mice were included into the passage.

### RNA extraction and cDNA preparation

Total RNA was extracted from 100 µL of a 10% brain or plasma suspension in PBS using Trizol LS (Life Science, USA) according to the manufacturer's recommendations. Then, RNA was precipitated, washed twice using 1 mL of 80% ethanol and air-dried for 5 min. The RNA precipitate was resuspended in 50 µL of fresh Milli-Q water and used as a template in a reverse transcriptase reaction as soon as possible. To prepare cDNA by reverse transcription, a mixture of 5 µL of RNA, 50 pM of hexanucleotide primers, 200 mM of each NTP, 5 mM MgCl_2_, 200 U of MMLV RT (Promega, Madison, WI) and Milli-Q water (up to a total volume of 50 µL) was incubated at 42°C for 30 min. Samples of cDNA for transportation were mixed with equal volumes of isopropanol and stored as a suspension.

### Amplification

Pairs of oligonucleotide primers for amplification were designed based on previously published sequences for the Sofjin, Senzhang, 205, Glubinnoe and Aina strains and optimized to generate 38 overlapping fragments for sense and antisense strains with an average length of about 1000 base pairs (Table S2). To amplify each fragment, a mixture of 0.5 µL of cDNA, 50 pM of the corresponding sense and antisense PCR primers, 25 µL of 2× PCR Mix (Fermentas, Lithuania) and fresh Milli-Q water (up to a total volume of 50 µL) was incubated at 95°C for 1 min and then used in a cycle sequencing reaction (25 cycles of 96°C for 5 sec, 57°C for 5 sec and 72°C for 30 sec). Amplicons were analyzed by electrophoresis in 1× TAE buffer on a 0.75% agarose gel (Promega, USA). Bands were excised from the gel under a UV transilluminator and DNA was eluted from the gel via the freeze-thawing method [96]. The amount of eluted DNA was analyzed using Nano View (GE Healthcare, USA).

### Sequencing of PCR fragments

For each sequencing reaction, approximately 50 to 100 ng of purified dsDNA were mixed with 3.2 pmol of one of the primers and with a reaction cocktail containing the four dye-labeled dideoxynucleotide terminators (BigDye V 3.1, Life Science, USA). The cycle sequencing parameters used were as described in the manufacturer's protocol (25 cycles of 96°C for 30 s, 50°C for 60 s and 60°C for 4 min). Reaction products were precipitated by ethanol-acetate as described in the manufacturer's recommendations. The pellet was resuspended in 16 µL of template suppression reagent, heated for 2 min at 95°C, and kept on ice until it was loaded into the Applied Biosystems Prism 3100 sequencer.

### Nucleotide and amino acid sequence analysis

Overlapping nucleic acid sequences were combined for analysis and edited with a software package (*BioEdit* v.7.2.0 sequence alignment editor) (http://www.mbio.ncsu.edu/bioedit/bioedit.html). The nucleotide sequence was translated into the amino acid sequence online at the Bioinformatics Resource Portal (http://web.expasy.org/translate/). The nucleotide and translated amino acid sequences of the strains were deposited in the international database GenBank (http://www.ncbi.nlm.nih.gov/) under the accession numbers shown in Table 2.

TBEV complete genomes and the predicted amino acid sequences were compared online at mafft.cbrc.jp/alignment server/ by the program MAFFT version 7 with the Sofjin-HO strain as the prototype TBE virus.

### Phylogenetic analyses

Phylogenetic analysis of nucleotide and amino acid sequences of the complete genomes of the analyzed virus strains and the previously reported genome sequences of the TBEV strains of Far East subtypes (Sofjin - AB062064, Oshima 5–10 - AB062063, Glubinnoe - DQ862460 and Senzhang - AY182009), the Vasilchenko strain as the prototype strain of the Siberian subtype, and Neudoerfl as the prototype strain of the Western subtype was performed using the Maximum Likelihood (ML) method. The phylogenetic tree was estimated using the ML method TreeFinder [97]. The best-fit evolutionary model was determined using jModelTest version 0.1 [98], and was General Time Reversible (GTR) with a gamma-distribution model of among-site rate heterogeneity and a proportion of invariant sites (GTR+C+I). The inferred trees were visualized using FigTree online version 1.12 (http://tree.bio.ed.ac.uk/software/figtree/)

Synonymous (dS) and non-synonymous (dN) substitution rates were calculated by CodeML in the PAML package [99], [100]

### Secondary structure analysis of the UTRs

RNA secondary structure predictions of the UTRs of viral RNA were performed using the mfold program, which is available from http://mfold.rna.albany.edu [101]. RNA hybrids of the first 240 nucleotides, including the 5′ UTR, part of the capsid protein gene, and a full-length 3 ′UTR, were used to predict secondary structure motifs and identify possible cyclization motifs in RNA. Secondary structure analysis of possible cyclization motifs was performed for all 34 TBEV sequences.

### Signal prediction

Prediction of the location and the possibility of signal sequence cleavage were carried out online at the SignalP 4.1 Server (http://www.cbs.dtu.dk/services/SignalP/) [102].

### Transmembrane domain prediction

Prediction of the transmembrane protein topology of viral proteins was performed online using hidden transmembrane helices at http://www.cbs.dtu.dk/services/TMHMM/TMHMM2.0b.guide.php [103], [104].

### Homology modeling

The tertiary structures of the TBEV NS2B-NS3 protease, the capsid protein C, and RdRP for the Dalnegorsk and Primorye-270 strains were modeled by the Nest program of the Jackal package [105], [106].

### NS2B/NS3 protease

The crystal structure of the West Nile protease (PDB code 3e90) was taken as a template for homology modeling as this model has a high resolution (2.45 Å) and the sequence identity between the target and the template is greater than 25% [107].

### Capsid protein C

The crystal structure of the capsid protein C from West Nile Virus, subtype Kunjin (PDB code 1sfk) was taken as a template for homology modeling [108].

### RdRP

The crystal structure of the dengue virus RNA-dependent RNA polymerase (RdRP) catalytic domain, at a resolution of 1.85 Å (PDB code 2j7u), was chosen as a template [109].

### Molecular dynamics simulations

The molecular dynamics (MD) simulations were performed using Amber software version 11. The Amber 99 force field was applied for the complexes [110]. Each protein complex was hydrated by 9 Å of the Simple Point Charge water model [111], applying the triclinic periodic box with an 8 Å cut-off point for the summation of the non-bonded interactions in the real space. Counter ions, Na+ or Cl-, were added by replacing the water molecules in each system in order to neutralize the system. Subsequently, 1000 iterations were applied to minimize the energy of the system. The position of the backbone atoms was restrained during the first 100 ps with a force constant of 2.0 kcal/Å^2^ in order to allow adjustment of the solvent molecules. After this, free molecular dynamics simulations were performed using the NPT (isothermal-isobaric) ensemble by applying a time step of 2 fs. The temperature was kept constant at 300 K. The Berendsen pressure control scheme was used to keep the pressure in the vicinity of 1 bar during the simulation with a relaxation time of 2 ps and a default compressibility parameter for water equal to 44.6×10^−6^ bar ^−1^. The system was considered equilibrated after 1 ns. The MD simulation without restraints was carried out for 20 ns for both complexes of NS2b/NS3, for 5 ns for both complexes of capsid protein C, and for 5 ns for both complexes of RdRP.

## Supporting Information

Figure S1 **The scheme of the 3′ UTR and the position of deletions.**
(TIF)

Figure S2 **The similarity matrix of genomes and polyproteins.** Pathogenic strains are shown in bold font and strains with the febrile form of TBEV are shown in red.
(TIF)

Figure S3 **Predicted secondary structure of Sofjin strain 5′ UTR.**
(TIF)

Figure S4 **Predicted secondary structures 3′ UTR of Sfd strain Primorye – 320 and Efd strain Svetlogorie.** Circles indicate random nucleotide substitutions, and squares indicate characteristic substitutions that differ from groups of Efd and Sfd strains.
(TIF)

Figure S5 **Predicted secondary structures of the interaction between at the 5′ and 3′ ends of the RNA of the TBE virus pathogenic strain Svetlogorie.** 5′ and 3′ flanking sequences connected by a RNA circle insert; the AUG initiation codon is boxed. The constant loop SLA and SLC and the new UAR (Upstream AUG) region are shown.
(TIF)

Figure S6 **Amino acid residue substitutions and positions in viral proteins.** The key amino acid residues in Sfd strains differ from the amino acid residues of pathogenic strains, indicated in bold and in gray blocks. The strains that cause disease of varying severity are color-coded according to the legend in Figure 1.
(TIF)

Figure S7 **A map of Primorye (Far East Russia) showing the location of sites in which patients were bitten by ticks.** Isolated Efd strains are shown in black, Sfd strains in green and strains with the febrile form of TBEV are shown in red.
(TIF)

Figure S8 **Prediction of the most probable C-prM signalase cleavage sites.** (A) Efd strain Svetlogorie, highlighted red square amino acid Leu that deleted in Sfd strains. (B) Sfd strain Primorye-320.
(TIF)

Figure S9 **Predicted tertiary structure of the capsid protein.** (A) The tertiary structure of the capsid protein dimer for the pathogenic strain Dalnegorsk. (B) The tertiary structure of the capsid protein dimer for the Sfd strain Primorye – 270.
(TIF)

Figure S10 **Transmembrane domains predictions in Efd strain Svetlogorie and Sfd strain Primorye – 320.** (A) Capsid proteins. (B) prM proteins. (C) Envelope proteins. (D) NS2B proteins. (E) NS4B proteins. Amino acid positions that differ in the Efd and Sfd strains are identified by colored lines.
(TIF)

Figure S11 **Aligned amino acid sequences of the flavivirus RNA polymerase domain.** Designations: TBEV – tick-borne encephalitis virus, strain Dalnegorsk, accession: FJ402886; OHFV - Omsk hemorrhagic fever virus, strain Guriev, accession: BAH78736; KFDV - Kyasanur forest disease virus, accession: AAQ91607; POWV - Powassan virus, strain Nadezdinsk-1991, accession: ACD88752; YFV - Yellow fever virus, strain Uganda 2010, accession: AEQ35299; DV2 - Dengue virus 3, isolate DENV-3/US/BID-V1473/2002, accession: ACD13417; DV2 - Dengue virus 2, strain D2/TO/UH39/1974, accession: ADM26233; JEV - Japanese encephalitis virus, isolate SC0415, accession: AEO72405; WNV - West Nile virus, accession: ADZ96248.
(TIF)

Table S1 **Results of prediction signal peptide cleavage.**
(DOCX)

Table S2 **List of primers used for amplification and sequencing of complete genomes.**
(DOCX)
