# Supplementary figures and images for: The Relationship between the Structure of the Tick-Borne Encephalitis Virus Strains and Their Pathogenic Properties
Source: PLoS One. 2014 Apr 16;9(4):e94946. doi: 10.1371/journal.pone.0094946 (PMC3989262; doi:10.1371/journal.pone.0094946)

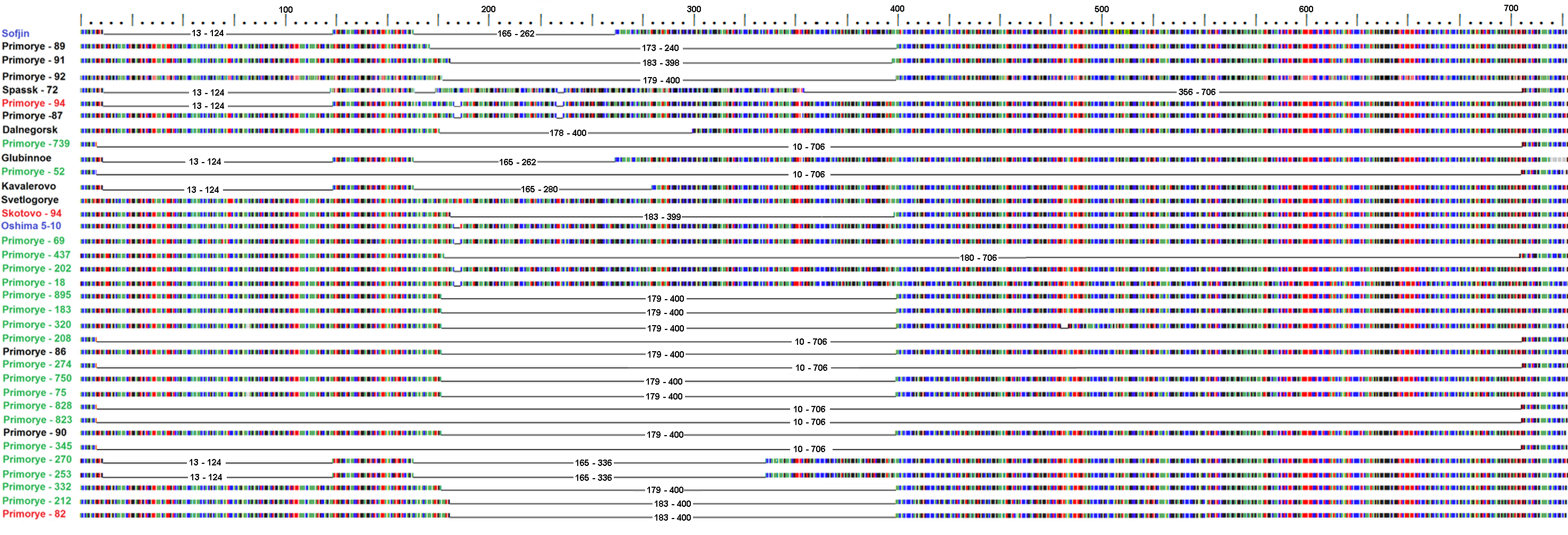

Supplement: Figure S1 — The scheme of the 3′ UTR and the position of deletions. (TIF) [file pone.0094946.s001.tif]

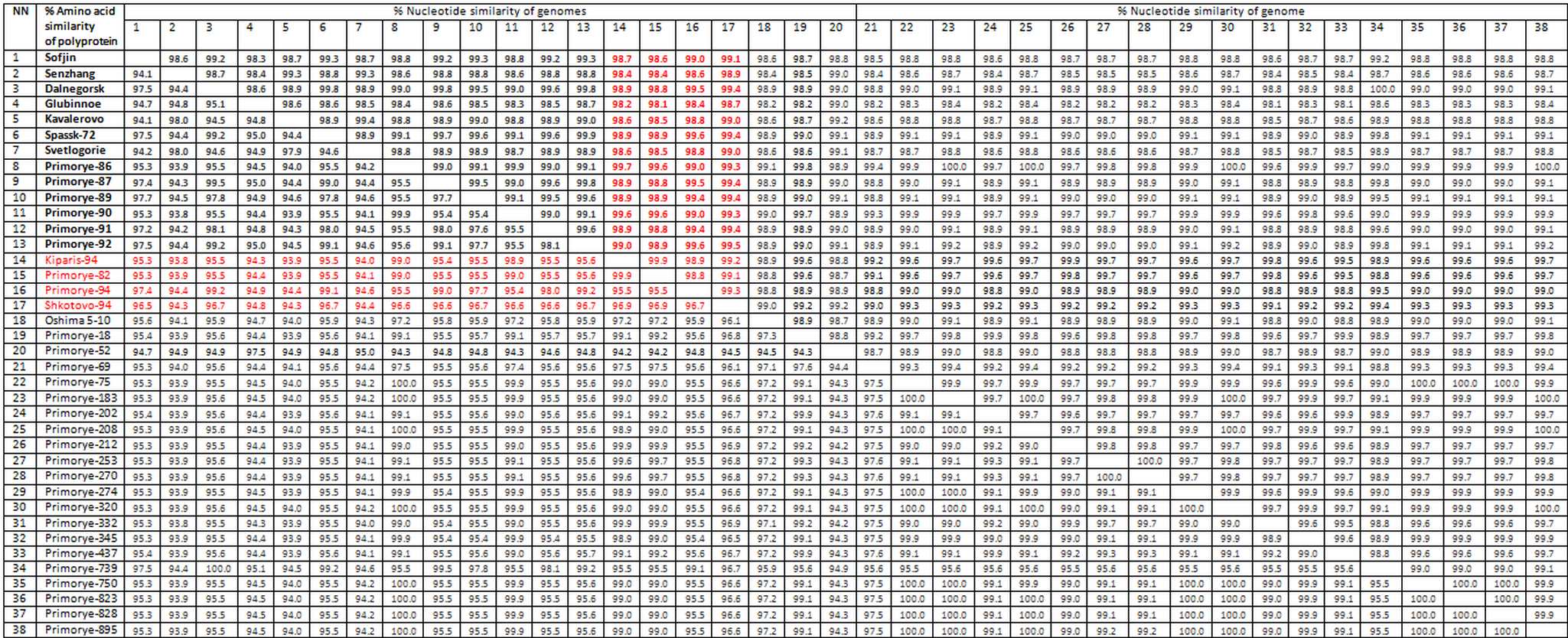

Supplement: Figure S2 — The similarity matrix of genomes and polyproteins. Pathogenic strains are shown in bold font and strains with the febrile form of TBEV are shown in red. (TIF) [file pone.0094946.s002.tif]

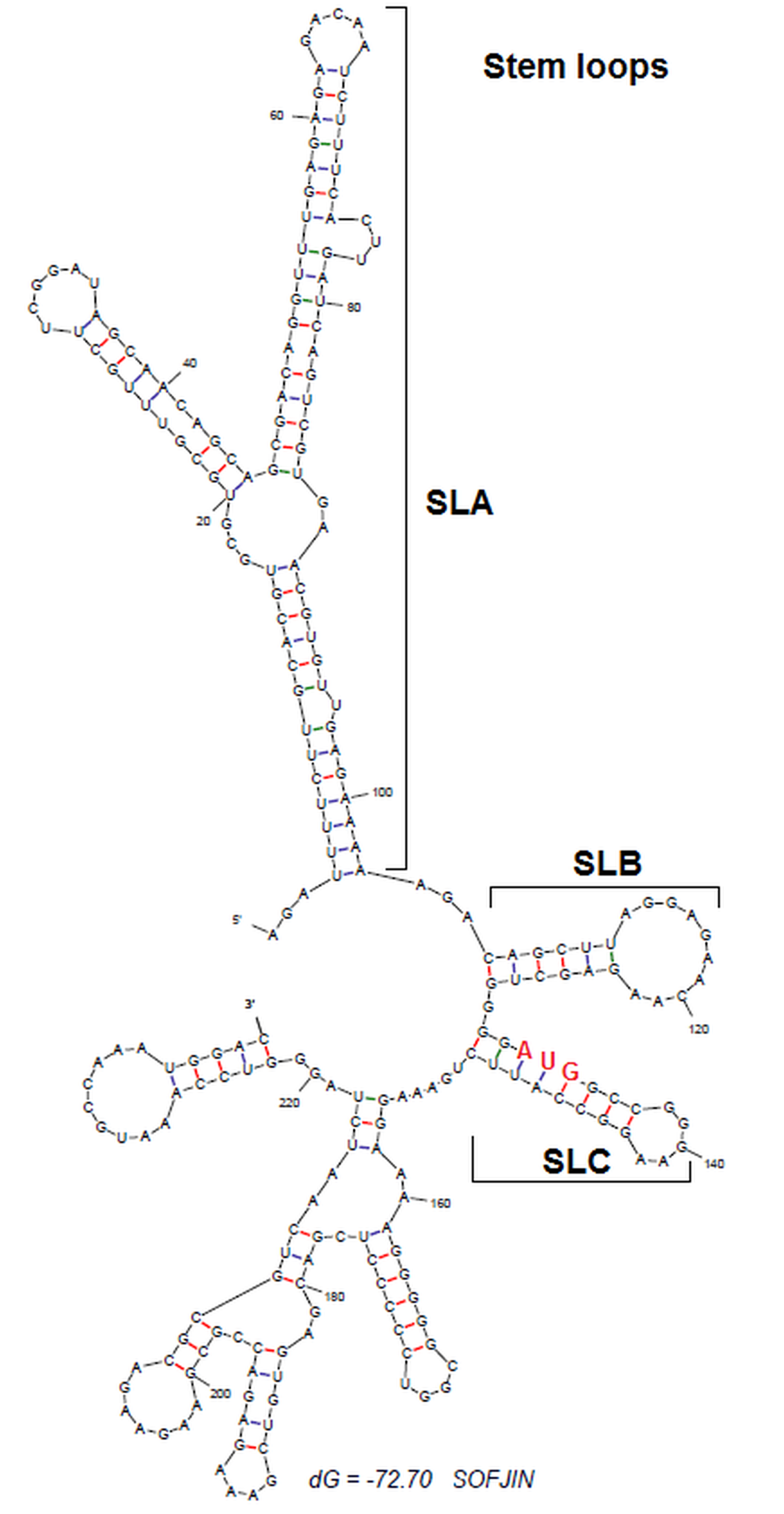

Supplement: Figure S3 — Predicted secondary structure of Sofjin strain 5′ UTR. (TIF) [file pone.0094946.s003.tif]

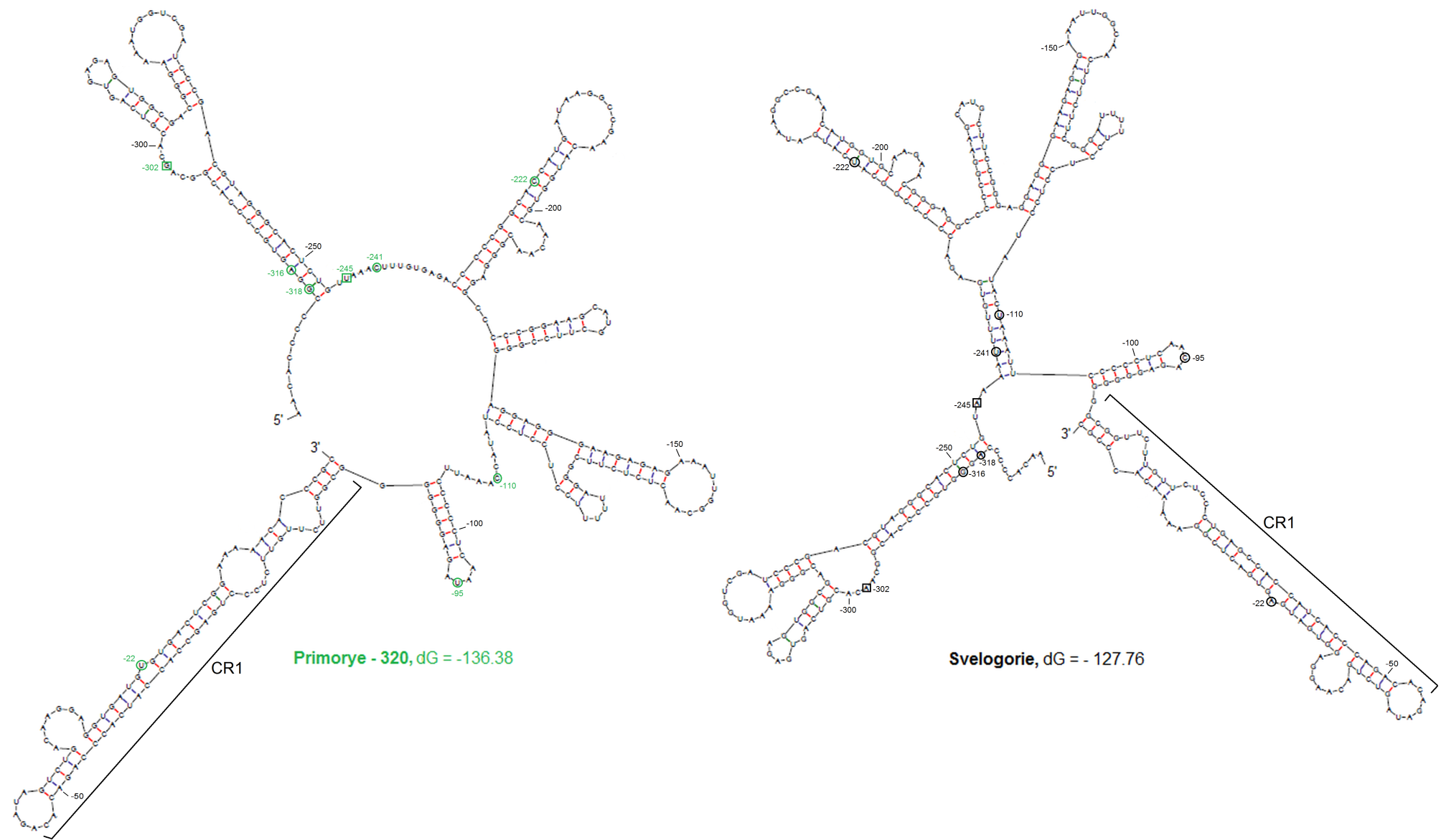

Supplement: Figure S4 — Predicted secondary structures 3′ UTR of Sfd strain Primorye – 320 and Efd strain Svetlogorie. Circles indicate random nucleotide substitutions, and squares indicate characteristic substitutions that differ from groups of Efd and Sfd strains. (TIF) [file pone.0094946.s004.tif]

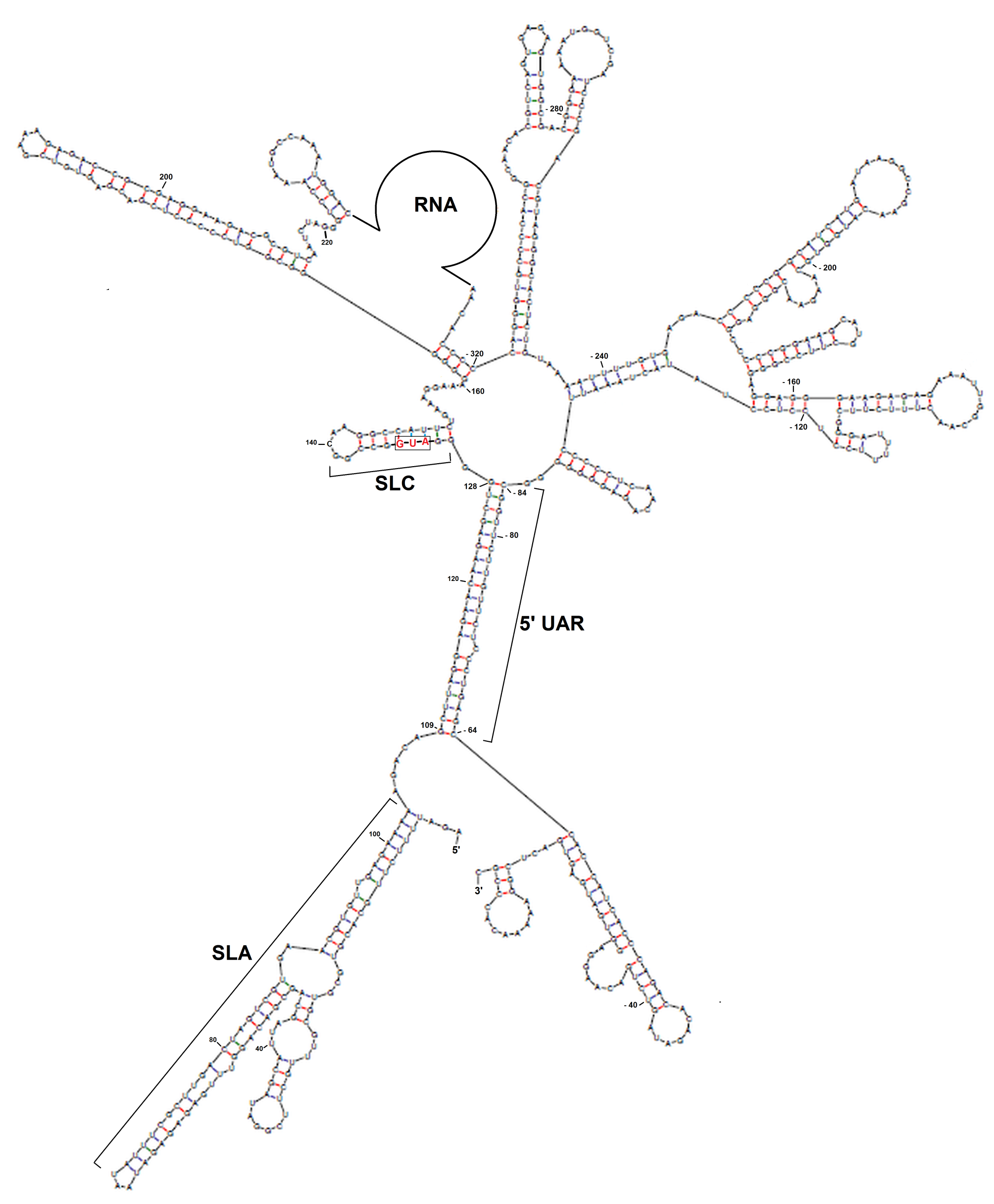

Supplement: Figure S5 — Predicted secondary structures of the interaction between at the 5′ and 3′ ends of the RNA of the TBE virus pathogenic strain Svetlogorie. 5′ and 3′ flanking sequences connected by a RNA circle insert; the AUG initiation codon is boxed. The constant loop SLA and SLC and the new UAR (Upstream AUG) region are shown. (TIF) [file pone.0094946.s005.tif]

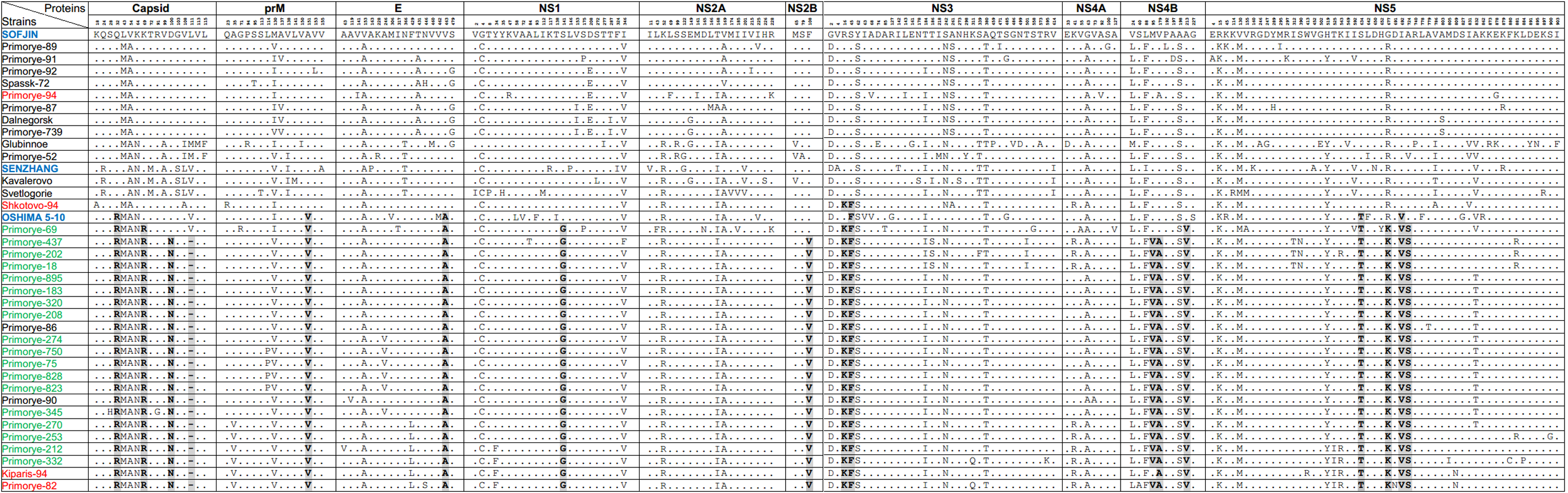

Supplement: Figure S6 — Amino acid residue substitutions and positions in viral proteins. The key amino acid residues in Sfd strains differ from the amino acid residues of pathogenic strains, indicated in bold and in gray blocks. The strains that cause disease of varying severity are color-coded according to the legend in Figure 1. (TIF) [file pone.0094946.s006.tif]

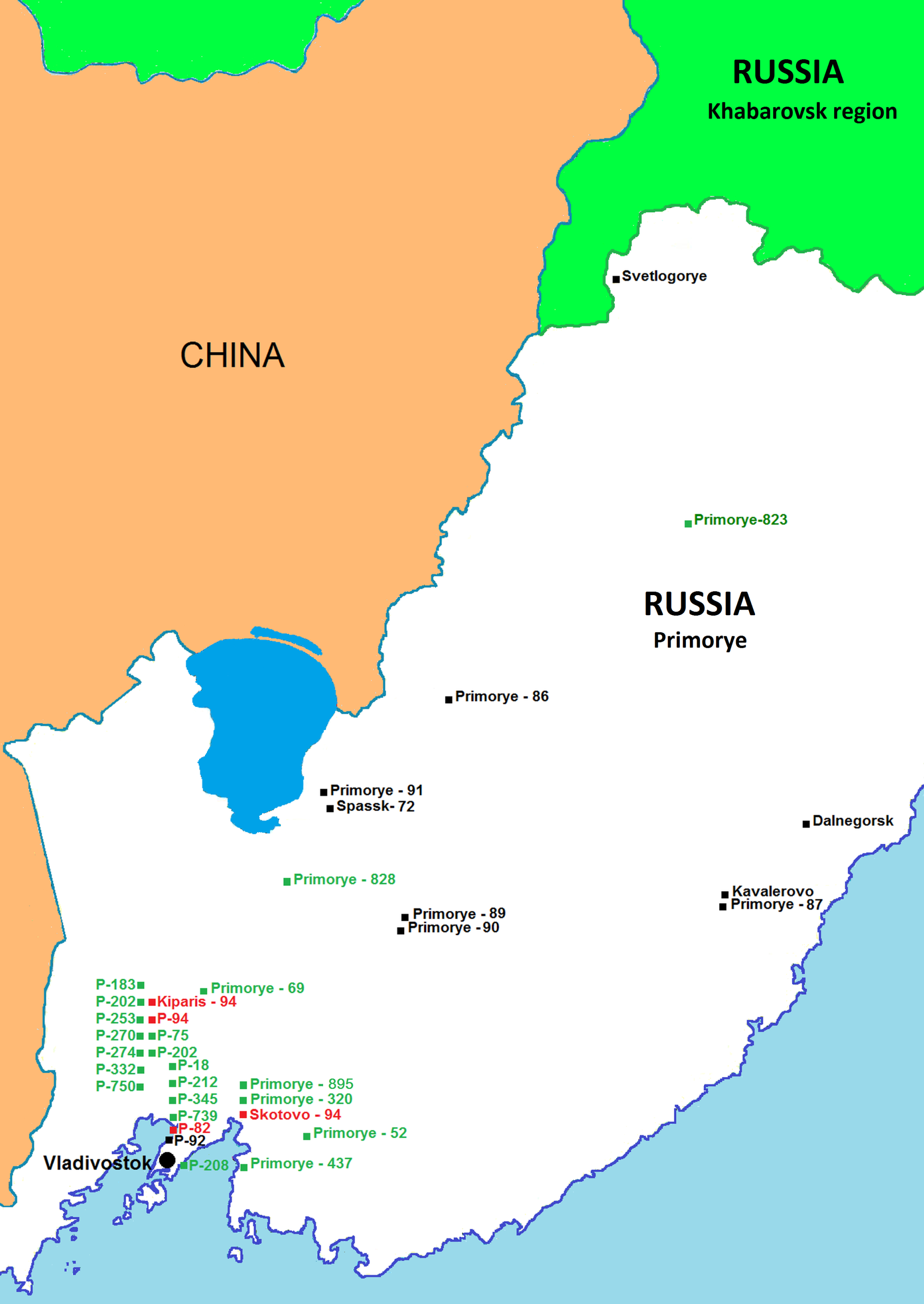

Supplement: Figure S7 — A map of Primorye (Far East Russia) showing the location of sites in which patients were bitten by ticks. Isolated Efd strains are shown in black, Sfd strains in green and strains with the febrile form of TBEV are shown in red. (TIF) [file pone.0094946.s007.tif]

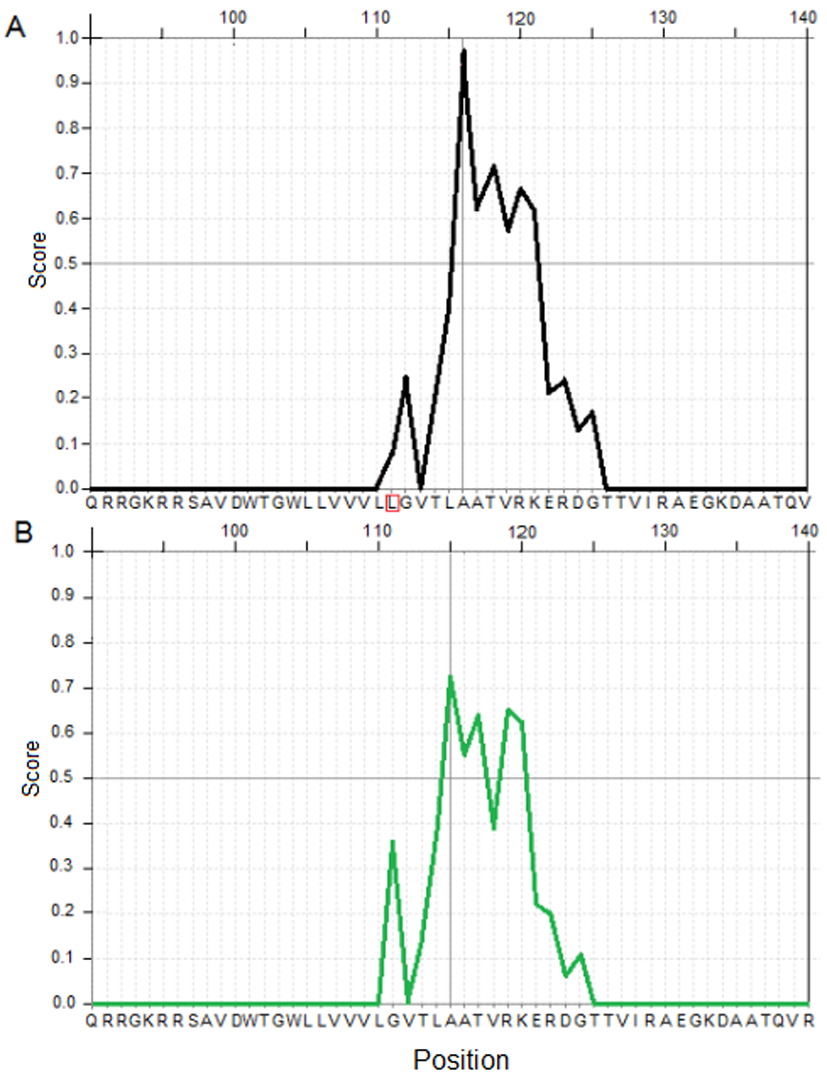

Supplement: Figure S8 — Prediction of the most probable C-prM signalase cleavage sites. (A) Efd strain Svetlogorie, highlighted red square amino acid Leu that deleted in Sfd strains. (B) Sfd strain Primorye-320. (TIF) [file pone.0094946.s008.tif]

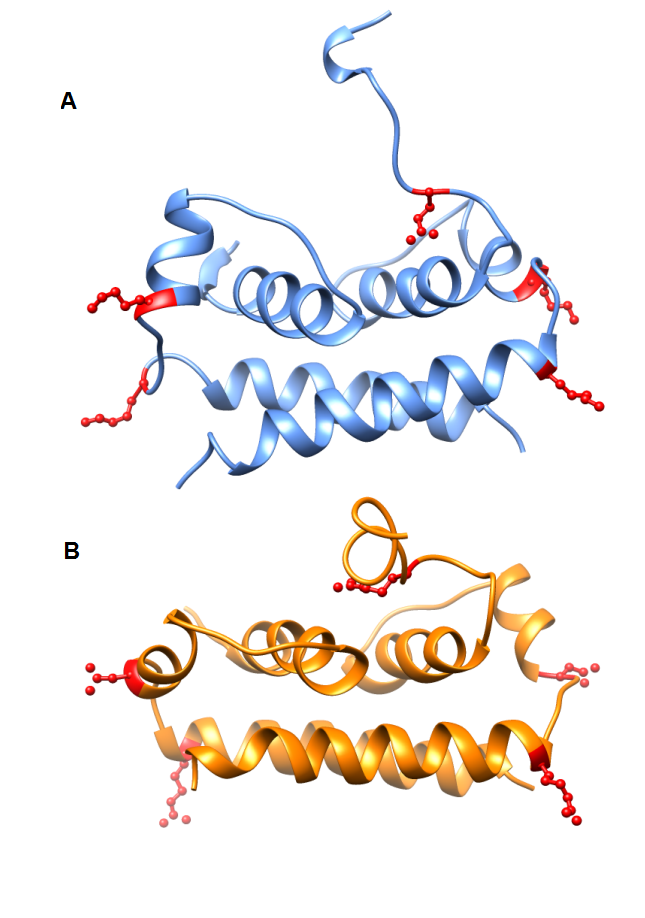

Supplement: Figure S9 — Predicted tertiary structure of the capsid protein. (A) The tertiary structure of the capsid protein dimer for the pathogenic strain Dalnegorsk. (B) The tertiary structure of the capsid protein dimer for the Sfd strain Primorye – 270. (TIF) [file pone.0094946.s009.tif]

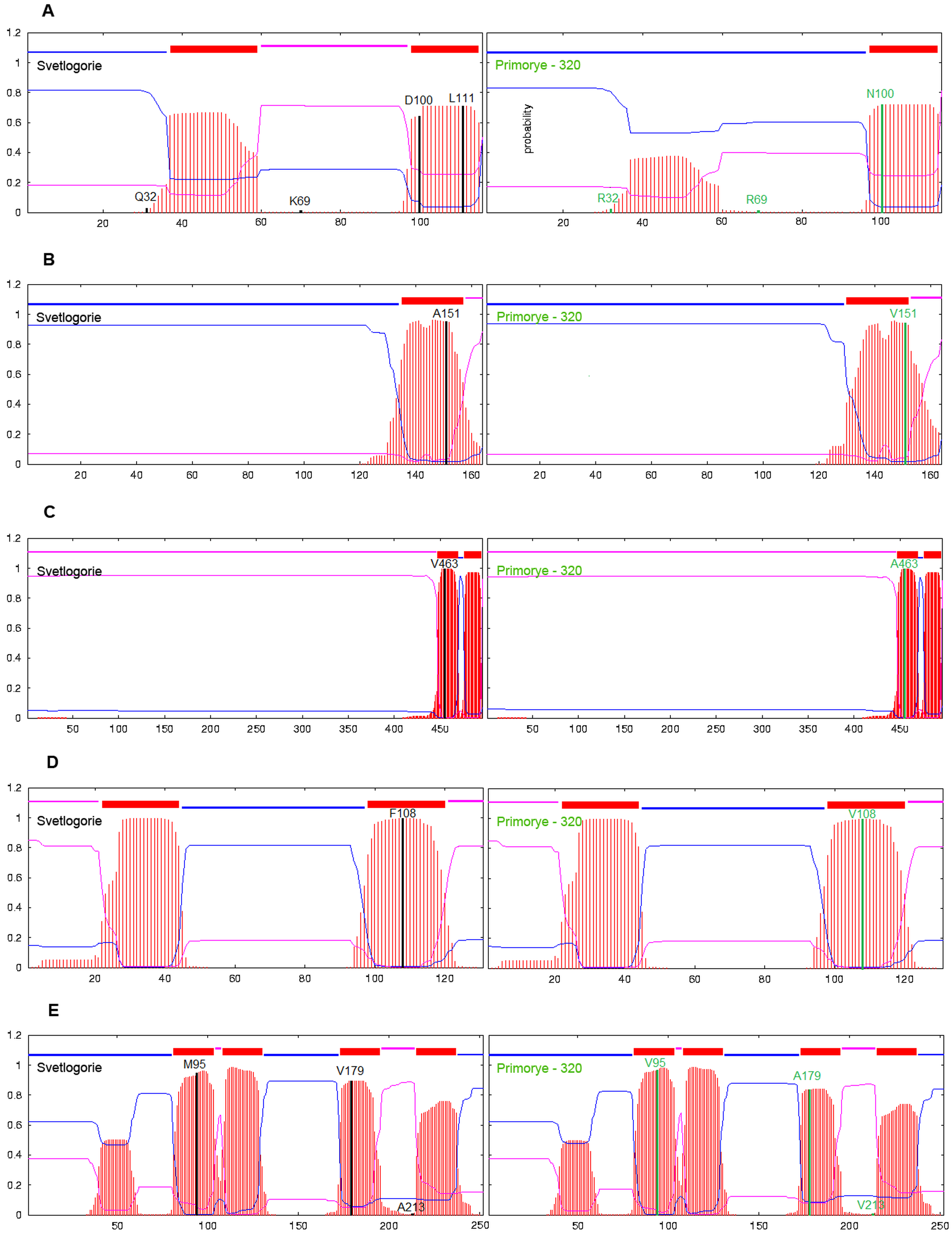

Supplement: Figure S10 — Transmembrane domains predictions in Efd strain Svetlogorie and Sfd strain Primorye – 320. (A) Capsid proteins. (B) prM proteins. (C) Envelope proteins. (D) NS2B proteins. (E) NS4B proteins. Amino acid positions that differ in the Efd and Sfd strains are identified by colored lines. (TIF) [file pone.0094946.s010.tif]

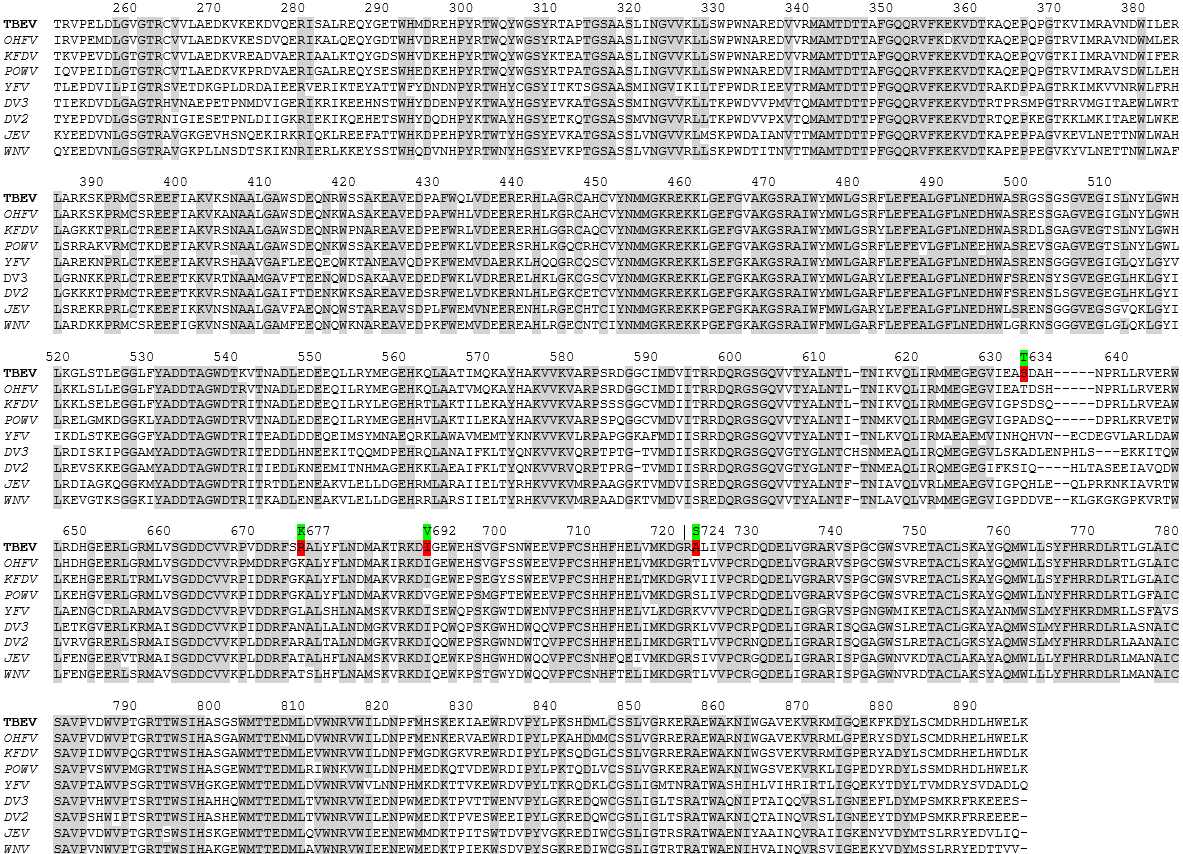

Supplement: Figure S11 — Aligned amino acid sequences of the flavivirus RNA polymerase domain. Designations: TBEV – tick-borne encephalitis virus, strain Dalnegorsk, accession: FJ402886; OHFV - Omsk hemorrhagic fever virus, strain Guriev, accession: BAH78736; KFDV - Kyasanur forest disease virus, accession: AAQ91607; POWV - Powassan virus, strain Nadezdinsk-1991, accession: ACD88752; YFV - Yellow fever virus, strain Uganda 2010, accession: AEQ35299; DV2 - Dengue virus 3, isolate DENV-3/US/BID-V1473/2002, accession: ACD13417; DV2 - Dengue virus 2, strain D2/TO/UH39/1974, accession: ADM26233; JEV - Japanese encephalitis virus, isolate SC0415, accession: AEO72405; WNV - West Nile virus, accession: ADZ96248. (TIF) [file pone.0094946.s011.tif]
