# Supplementary material for: The Relationship between the Structure of the Tick-Borne Encephalitis Virus Strains and Their Pathogenic Properties
Source: PLoS One. 2014 Apr 16;9(4):e94946. doi: 10.1371/journal.pone.0094946 (PMC3989262; doi:10.1371/journal.pone.0094946)
Supplement: Table S1 — Results of prediction signal peptide cleavage. (DOCX) [file pone.0094946.s012.docx]

**Table S1. The results of the prediction signal peptide cleavage**

|  | Sofjin | The hypothetical variant 1 | Primorye-320 | The hypothetical variant 2 |
| --- | --- | --- | --- | --- |
| max. C | 0.611 | 0.256 | 0.427 | 0.684 |
| max. S | 0.767 | 0.723 | 0.981 | 0.795 |
| max. Y | 0.584 | 0.371 | 0.633 | 0.670 |

C - cleavage site score;

S - signal peptide score;

Y - combined cleavage site score

Amino acid sequences used to calculate:

strain Sofjin

QRRGKRRSAV**D**WTGWLLVVVL**L**GVTLAATVRKERDGTTVIRAEGKDAATQVR,

the hypothetical variant 1

QRRGKRRSAV**D**WTGWLLVVVL-GVTLAATVRKERDGTTVIRAEGKDAATQVR,

strain Primorye-320

QRRGKRRSAV**N**WTGWLLVVVL-GVTLAATVRKERDGTTVIRAEGKDAATQVR,

the hypothetical variant 2

QRRGKRRSAV**N**WTGWLLVVVL**L**GVTLAATVRKERDGTTVIRAEGKDAATQVR
