# Supplementary material for: The Relationship between the Structure of the Tick-Borne Encephalitis Virus Strains and Their Pathogenic Properties
Source: PLoS One. 2014 Apr 16;9(4):e94946. doi: 10.1371/journal.pone.0094946 (PMC3989262; doi:10.1371/journal.pone.0094946)
Supplement: Table S2 — List of primers used for amplification and sequencing of complete genomes. (DOCX) [file pone.0094946.s013.docx]

**Table S1. List of primers used for amplification and sequencing of complete genomes**

| Name | Designaton^a^ | Sequence (5’→3’) | Position^b^ |
| --- | --- | --- | --- |
| 1 | 1F | AGATTTTCTTGCACGTGCGTGCG | 1-23 |
| 2 | 500F | CCGTGTTGAAGTCTTTCTGGAA | 301-322 |
| 3 | 500R | GTGAGTCATCACACCATGATCC | 615-594 |
| 4 | 1000F | CTGGCTCCGGTTTATGCCTCAC | 954-975 |
| 5 | 1000R | ACGCATCCTCCCAGTTCCAG | 1063-1044 |
| 6 | 1500F | CCTGTGTCAAGGCGTCTTGTG | 1315-1335 |
| 7 | 1500R | ACTCCAGTCTGGTCTCCAAGG | 1759-1739 |
| 8 | 2000F | GCGTTCTCTGGGACCAAACCC | 1962-1983 |
| 9 | 2000R | TTGGGTGTTATCAACATGGCCAC | 2052-2031 |
| 10 | 2500F | ATGAGGAATCCGACCATGTCC | 2382-2402 |
| 11 | 2500R | AGTTCCCTCCTCGAAGGTCTC | 2621-2601 |
| 12 | 3000F | GGACTTCAGACAGGAATCAAC | 2966-2986 |
| 13 | 3000R | GGTCTGTGTGGACTGCCATGCC | 3051-3030 |
| 14 | 3500F | TGAAAGGACCATGGAAGTACTC | 3253-3274 |
| 15 | 3500R | TTCCAGTGGCTGGCCTCCTC | 3612-3593 |
| 16 | 4000F | GGTGTTGGACTTCTGCTCATGG | 3969-3990 |
| 17 | 4000R | CAACATGAGGCCACGCCC | 4063-4046 |
| 18 | 4500F | CATTCAGTGAACCACTGACTG | 4207-4227 |
| 19 | 4500R | CGTCCACAATCCCATCACAC | 4568-4549 |
| 20 | 5000F | GAACTGCTCCTGGACACAGGT | 4947-4967 |
| 21 | 5000R | ACCACTCCCTGGGAGTTGAGG | 5044-5024 |
| 22 | 6000F | CACAGGGACCAGACGTGTGAC | 5936-5956 |
| 23 | 6000R | TCATCATCACACTGTCCTGAG | 6046-6026 |
| 24 | 6500F | CGTGTCTAGTGTGACAAGTCG | 6263-6283 |
| 25 | 6500R | CTGCCAGGCTCCTCATGCATC | 6571-6551 |
| 26 | 7000F | CGGTGTTGTGGAGTGAACATG | 6952-6972 |
| 27 | 7000R | TGGTGTGAACAGGGAGACCAC | 7067-7047 |
| 28 | 7500F | AAGTCTTCTTCTCGGCAATGG | 7321-7341 |
| 29 | 7500R | CACAGGCCACTGGCATCGTC | 7578-7559 |
| 30 | 8000F | ATGTGGAAGAGGCGGCTGGTC | 7907-7927 |
| 31 | 8000R | GCTCTGAACTTGATCAGGTTC | 8047-8027 |
| 32 | 8500F | GCGGAGGACTGGTGAGGAC | 8275-8294 |
| 33 | 8500R | AGTACTGCCAGGTCCTGTACG | 8583-8563 |
| 34 | 9000F | TGAGCAGAACAGGTGGTCAAG | 8909-8929 |
| 35 | 9000R | GTACCATATGGCCCGGCTTCC | 9095-9075 |
| 36 | 9500F | AGGGTGAACACAAGCAACTGG | 9340-9361 |
| 37 | 9500R | TCCGAGACGTTCTTCTCCATG | 9615-9635 |
| 38 | 10000F | TCCGTGAAACTGCCTGCCTTTC | 9910-9930 |
| 39 | 10000R | GTCCTCTGTGGTCATCCAGGC | 10091-10071 |
| 40 | 11000R | GCGGGTGTTTTTCCGAGTCAC | 10893-10873 |

^a^Designaton with an F indicate a viral-sense orientation, with R indicate a reverse orientation.

^b^Genome positions are given according to the published sequence of strain Sofjin-HO
